# Emerging Trends in Phosphorene Fabrication towards Next Generation Devices

**DOI:** 10.1002/advs.201600305

**Published:** 2017-02-07

**Authors:** Sathish Chander Dhanabalan, Joice Sophia Ponraj, Zhinan Guo, Shaojuan Li, Qiaoliang Bao, Han Zhang

**Affiliations:** ^1^ SZU‐NUS Collaborative Innovation Center for Optoelectronic Science and TechnologyKey Laboratory of Optoelectronic Devices and Systems of Ministry of Education and Guangdong ProvinceCollege of Electronic Science and Technology, and College of Optoelectronics EngineeringShenzhen UniversityShenzhen518060China; ^2^ Institute of Functional Nano and Soft Materials (FUNSOM)Jiangsu Key Laboratory for Carbon‐Based Functional Materials and Devices, and Collaborative Innovation Center of Suzhou Nano Science and TechnologySoochow UniversitySuzhou215123P. R. China; ^3^ Department of Nanoscience and TechnologyBharathiar UniversityCoimbatore‐641046TamilnaduIndia; ^4^ Department of Materials Science and EngineeringMonash UniversityWellington RoadClaytonVictoria3800Australia

**Keywords:** black phosphorus, exfoliation, fabrication, passivation, phosphorene, two‐dimensional materials

## Abstract

The challenge of science and technology is to design and make materials that will dominate the future of our society. In this context, black phosphorus has emerged as a new, intriguing two‐dimensional (2D) material, together with its monolayer, which is referred to as phosphorene. The exploration of this new 2D material demands various fabrication methods to achieve potential applications— this demand motivated this review. This article is aimed at supplementing the concrete understanding of existing phosphorene fabrication techniques, which forms the foundation for a variety of applications. Here, the major issue of the degradation encountered in realizing devices based on few‐layered black phosphorus and phosphorene is reviewed. The prospects of phosphorene in future research are also described by discussing its significance and explaining ways to advance state‐of‐art of phosphorene‐based devices. In addition, a detailed presentation on the demand for future studies to promote well‐systemized fabrication methods towards large‐area, high‐yield and perfectly protected phosphorene for the development of reliable devices in optoelectronic applications and other areas is offered.

## 1 Introduction

As a result of the interest brought about by the superior optical and electronic properties of graphene, two‐dimensional (2D) materials have been extensively studied in recent years due to their widespread applications in the field of novel photonic devices, including photodetectors, optical absorbers, optical modulators, and fiber lasers.^1^, ^2^ Most of the applications currently being developed with 2D materials are particularly pertinent because they directly address many of the scientific challenges confronting the modern world. These materials, as a result of their self‐limiting nature, have emerged as candidates with a great capability to move present research from the nanometer scale to the 2D regime by supplementing innumerable research opportunities to allow exploration of the photonic applications based on them. The key factor of 2D materials lies on the ability of their band gap (*E*_g_) to be tuned based on the thickness of their layers so they exhibit either insulator or metal properties; this advantage can be employed in broadband photonic device applications.^3^, ^4^

However, so far the available 2D materials have either a very small band gap, from zero to about 0.3 eV (e.g., graphene and metallic dichalcogenides), or a reasonably large band gap, from 1 to 2 eV (e.g., semiconducting dichalcogenides). In this light, black phosphorous (BP), a new class of 2D layered materials with a layer‐dependent band gap ranging from 0.3 eV (bulk) to 1.5 eV (monolayer), can bridge the gap between the gapless graphene and large band gap transition metal dichalcogenides (TMDCs). Unlike few‐layer TMDCs, which mostly have an indirect band gap, few‐layer BP always has a direct bandgap for all thicknesses, a significant benefit for optoelectronic applications.^5^, ^6^, ^7^ Furthermore, the carrier mobility of phosphorene is significantly higher than other 2D materials including transition TMDCs. Phosphorene conducts electrons quickly, at a similar rate as that of graphene, but it has a considerable band gap, which is absent in graphene.^8^, ^9^, ^10^, ^11^, ^12^ The most striking property of layered BP is its in‐plane anisotropy, i.e., angular‐dependent optical conductivity and carrier mobility.^13^ One intriguing prospect is that heterostructures consisting of BP and another isotropic material may inherit anisotropic electrical and optical properties from BP, leading to new characteristics and possible optoelectronic applications. In addition, the strong resonant absorption of BP at infrared telecommunication wavelengths as well as its ultrafast carrier dynamics makes it an attractive saturable absorber for ultrafast laser photonics.^14^

It is noteworthy that BP is the only stable form of phosphorus that can be mechanically exfoliated in a similar manner as that of graphene and other 2D materials.^15^ BP can be produced from red phosphorous under high temperature and high pressure, where the layers (L) in BP are held by weak interlayer van der Waals forces.^5^ Remarkably, the layered black phosphorous (BP) material can be reduced to one single atomic layer in the vertical direction as a result of its van der Waals structure. The monolayer of BP, known as phosphorene, exhibits physical properties that can be significantly different from those of its bulk counterpart.^16^ Phosphorene has changed the landscape of many research areas in science and technology, particularly in condensed matter physics, and it has received much attention recently for its use as the base component of novel nanodevices, e.g., transistors, nanomechanical resonators, photovoltaics, photodetectors, batteries and sensors.^9^, ^10^, ^17^, ^18^, ^19^, ^20^, ^21^, ^22^, ^23^, ^24^, ^25^, ^26^, ^27^, ^28^, ^29^, ^30^, ^31^, ^32^, ^33^, ^34^, ^35^, ^36^, ^37^

The important challenge in realizing phosphorene devices is caused by its strong reaction with oxygen and water and so it should be well‐protected from degradation by encapsulating or sandwiching between different materials. According to a survey, 80% of the papers published in phosphorene are found to be at the theoretical level and there are many things as yet unexplored.^38^ The production of cheap, uniform, defect‐free layers and their simplicity are promising in the field of phosphorene device fabrication, and this forms the main scope of the present review.

**Figure 1:**
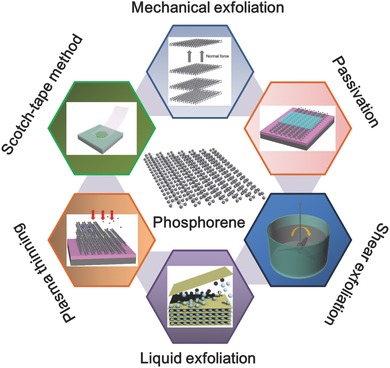
Schematic representation for the overview of the present review.

The rapidly increasing number of publications in the area of phosphorene research forms a strong motivation for the comprehensive review presented here. The presented review is organized with an emphasis on the fabrication of phosphorene, as it is the dominant topic in the area of 2D materials research. In this review we describe the recent progress in BP from the viewpoint of fabrication and the underlying mechanisms that are related to it. The review begins with an overview of the structure of phosphorene and continues by highlighting the properties of phosphorene. The production of phosphorene by different methods is discussed under two main topics: growth of bulk and few‐layered BP. Special attention is given to tabulating the experimental details with all growth parameters to benefit readers and researchers who are interested in this field. This review includes a detailed discussion of the key lessons learnt from the different fabrication techniques as well as the major difficulties encountered by researchers in implementing phosphorene in different device structures. **Figure** **1** gives a schematic representation of the overview of the review presented here.

## 2 Structure and Fundamentals of Phosphorene

In the past year, a new two‐dimensional material, i.e., BP has generated considerable excitement in the research community. Tremendous efforts are still ongoing to uncover the full potential of BP. Remarkably, the recent successful demonstration of phosphorene, the monolayer of BP, has given a renaissance to this material. The crystal structure of BP was first determined in 1935 from X‐ray diffraction studies of BP powder by Hultgren et al.^39^

### 2.1 Structure of Black Phosphorus and Phosphorene

**Figure 2:**
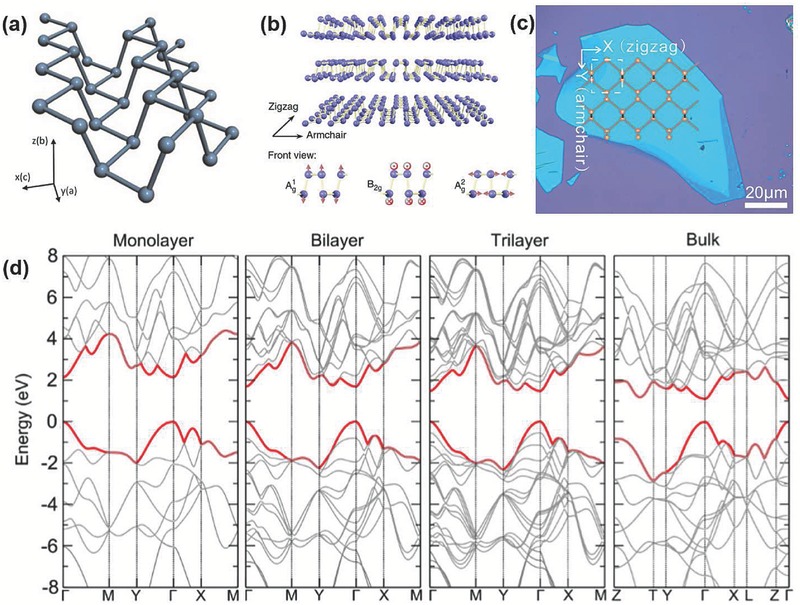
a) Three‐dimensional lattice structure of phosphorene. Reproduced with permission.^22^ Copyright 2014, American Institute of Physics. b) Lattice structure of BP with atomic vibrational patterns of the phonon modes (A_1g_, B_2g_ and A). Reproduced with permission.^148^ Copyright 2015, Nature Publishing Group. c) Crystalline orientation of 15L BP flake. Reproduced with permission.^63^ Copyright 2014, American Chemical Society. d) Calculated electronic band structure of monolayer, bilayer, trilayer and bulk BP in Brillouin zone in which the energy is scaled with respect to Fermi energy *E*_F_. Reproduced with permission.^156^ Copyright 2014, IOP.

**Figure 3:**
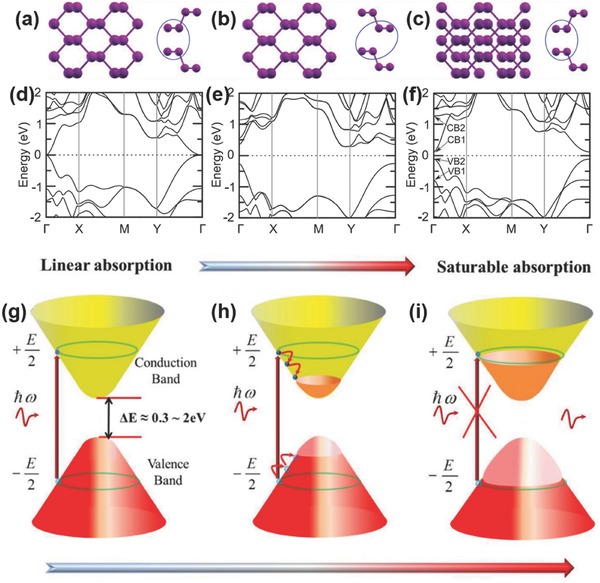
Top (left panel) and side (right panel) views of three stacking structures for bilayer BP: a) AC, b) AA and c) AB stacking. d–f) The corresponding band structures for the three stackings mentioned above. The Fermi level is set to 0 eV. The valence bands VB1 and VB2, as well as the conduction bands CB1 and CB2, are denoted in (f). Reproduced with permission.^55^ Copyright 2015, IOP. g–i) Schematic diagram of saturable absorption in multi‐layer BP nanoparticles. Reproduced with permission.^12^ Copyright 2015, The Optical Society of America.

Phosphorene has a puckered structure with reduced symmetry, in comparison to graphene, that gives rise to two anisotropic in‐plane directions.^40^ **Figure** **2** shows the lattice structure of phosphorene and the electronic band structures of monolayer, bilayer, trilayer and bulk BP. Due to the puckered configuration of phosphorene, anisotropy is observed in its optical properties,^41^, ^42^, ^43^ mechanical properties,^13^, ^44^, ^45^, ^46^ thermoelectric properties,^47^ electrical conductance^48^ and Poisson's ratio.^45^, ^49^, ^50^, ^51^ The high in‐plane anisotropic conductivity of few‐layer BP has also been reported.^41^ The quasi‐two‐dimensional puckered structure results in a highly anisotropic and nonlinear Young's modulus and ultimate strain.^46^ These anisotropic behaviors were found to be modulated with the biaxial or uniaxial strain imposed on BP, and the anisotropy could pave the way for realizing novel optoelectronic, electronic and nano‐mechanical devices using BP.^40^, ^45^, ^48^, ^52^, ^53^ The atomic layers of BP are stacked together by means of van der Waals interactions similar to those in graphene. Interestingly, single‐layer BP is covalently bonded with sp^3^ phosphorus atoms, where each phosphorus atom is covalently bonded to three neighboring phosphorus atoms with one lone pair of electrons, thereby forming a quadrangular pyramid‐shaped structure. Favron et al.^54^ designated the monolayer of exfoliated BP as stratophosphane or 2D‐phosphane instead of phosphorene, because the monolayer is made up of tervalent phosphorus atoms in agreement with the IUPAC nomenclature of the phosphane group. The top and side views of the three stacking structures AC, AA and AB for bilayer BP, together with their corresponding band structures, are presented in **Figure** **3**a–f. It is found that three stackings represent the stacking manner in their respective band structures where the decrease in the interlayer nearest P atoms distance cause the increase in interlayer Coulomb interactions. Hence, AC stacking has the maximum energy barrier at the time of sliding processes with comparatively harder exfoliation than the other sliding pathways.^55^

### 2.2 Properties

BP is a semiconductor with a direct band gap that is layer‐dependent and varies significantly between ≈2.2 eV in a monolayer and ≈0.3 eV in the bulk.^34^, ^41^, ^42^, ^56^, ^57^, ^58^, ^59^, ^60^, ^61^, ^62^, ^63^, ^64^, ^65^, ^66^ As a direct and narrow‐bandgap semiconductor, p‐type black phosphorus has significant advantages as a building block for functional optoelectronic devices.^56^, ^67^ Phosphorene monolayer (0.53‐nm thick) is a semiconductor with a direct band gap of 0.9 eV.^57^, ^68^ It has been reported that its *E*_g_ varies significantly between 0.9 eV in a monolayer and 0.1 eV in the bulk with the capability of tuning the electronic properties.^69^ The optical conductivity and optical absorption spectra of multi‐layer black phosphorus are reported to vary with the layer thickness, doping and light polarization at a frequency range of 2500 to 5000 cm^−1^.^43^ BP flakes have shown exciting electronic properties, as indicated by their hole mobility of 100 cm^2^ V^−1^ s^−1^ to 1000 cm^2^ V^−1^ s^−1^ with an on/off ratio of 10^2^ to 10^5^ at room temperature.^21^, ^22^, ^35^ As reflected from its structural anisotropy, BP has a larger carrier mobility than MoS_2_ and its photoresponsivity is larger than that of graphene.^21^, ^26^, ^34^, ^70^, ^71^ Combined with its high mobility, this shows potential for use in fast and broadband photodetector and solar cell applications.^24^, ^41^

Theoretical studies have shown that the in‐plane Young's modulus and ideal strain values of single‐layer BP are 41.3 GPa and 0.48, respectively, in the direction perpendicular to the pucker, and 106.4 GPa and 0.11, respectively, in the parallel direction.^46^ BP has the ability to withstand a tensile strain of up to 30% and 32% for monolayer and multi‐layer BP, respectively; the superior flexibility of phosphorene can also be utilized for practical large‐magnitude strain engineering.^46^, ^72^ The presence of a negative Poisson's ratio (ν = –0.027) in the out‐of‐plane direction under a uniaxial deformation in the direction parallel to the pucker in single‐layer BP was confirmed from first‐principles calculations.^49^ Due to the Hall mobility in the BP two‐dimensional electron gas, quantum oscillations at the extreme quantum limit are observed.^73^ Its Seebeck coefficient (*S*), measured at a temperature of 300 K to 385 K, is found to be *S* = +335 ± 10 µV K^–1^ at room temperature, which is evidence of a naturally occurring p‐type conductivity.^74^ Apart from its ability to tune the band gap between the valence band and conduction band local extrema, strain also plays a significant role in tuning the effective masses, thereby affecting the exciton anisotropy and binding strength.^75^ The properties of BP depends on the layer thickness,^42^ applied strain force,^45^, ^48^, ^52^, ^53^, ^64^, ^76^ stacking order^77^, ^78^ and external electric field,^79^ enabling the realization of devices for different applications such as electronics,^35^, ^37^, ^80^, ^81^, ^82^, ^83^ optoelectronics,^84^, ^85^, ^86^ energy storage,^79^, ^87^ saturable absorbers (SA),^2^, ^88^, ^89^, ^90^, ^91^, ^92^ pulsed lasers^93^, ^94^, ^95^ and sensing.^96^

In particular, the nonlinear optical property in terms of saturable absorption was observed in BP flake as well as its composites.^2^, ^90^, ^93^ Figure 3g–i depicts the schematic diagram of the saturable absorption in multi‐layer BP nanoparticles. A saturable absorber is a passive mode‐locking element for the generation of ultrashort pulses in solid‐state lasers. The mode‐locking and Q‐switching operation of fiber lasers based on BP is generously influenced by the saturable absorption parameters that form a major part of periodically modulating intracavity loss and managing the continuous‐wave laser into pulsed operations. BP‐based SAs with 648 fs, 940 fs, 786 fs and 272 fs mode‐locked pulses around 1.5 µm have been produced with the aid of Er‐doped fiber lasers showed maximum average output powers of 5.6 mW, 1.5 mW, 1 mW and 0.5 mW, respectively.^2^, ^91^, ^97^, ^98^ Similar to graphene‐based saturable absorbers,^99^, ^100^ absorption bleaching originates from Pauli blocking processes, in which a large number of photogenerated carriers cause band filling. A saturation intensity of 1.53 MW cm^–2^ can be obtained,^93^ which is comparable to those reported for graphene and semiconductor saturable absorber mirrors (SESAMs).^101^ The modulation depth of BP is found to be around 10.6%,^93^ comparable to that of carbon‐nanotube‐based saturable absorbers, which also have resonant absorption in the telecommunication bands.^102^ The advantages of BP as a saturable absorber lie in its strong resonant absorption at infrared telecom wavelenths as well as its ultrafast carrier dynamics, affording applications for ultrafast laser photonics.

## 3 Bulk growth of BP

The growing interest in phosphorene due to its great potential has increased the demand for large BP crystals for use in industrial applications.^27^ **Table** **1** depicts the different studies based on bulk growth of BP. BP in its bulk form can be synthesized through different methods such as the high‐pressure route,^35^, ^103^, ^104^, ^105^, ^106^, ^107^, ^108^ recrystallization from bismuth (Bi) flux,^59^, ^60^, ^109^ chemical vapor transport^110^, ^111^, ^112^, ^113^, ^114^ and mechanical milling.^115^, ^116^

**Table 1:** Studies on bulk growth of BP

Method	Parameters	Starting material	Final Product	Reference
High pressure	Pressure (≈11 000 to 13 000 kg cm^–2^), 200 °C	white phosphorus	black phosphorus	103
High pressure	10 kbar, 1000 °C cooled to 600 °C	red P	BP	35, 105
High pressure	10 kbar, 1000 °C cooled to 600 °C	red P	BP	108
HPHT	2 to 5 GPa	white P, red P	BP	107
HPHT	4 GPa, 800 °C, 10 min	red P	BP	118
Bi‐flux	quartz ampoule, Ar, 400 °C, 48 h	white P, red P (1 g), Bi (20 g)	BP	109
CVT	873 K for 10 h	red P (500 mg), AuSn (364 mg), SnI_4_ (10 mg)	BP	121
CVT	ampoule (10 cm length, 1.0 cm diameter, wall thickness of 0.25 cm), 7.5 h, 550 °C	red P (500 mg), Sn (20 mg), SnI_4_ (10 mg)	BP	122
CVT‐low pressure	silica glass ampoule (10 cm in length, 10 mm in diameter), 10^–3^ mbar, 873 K, 23 h	red P (500 mg), AuSn (364 mg), SnI_4_ (10 mg)	BP	112
CVT‐low pressure	silica glass ampoule (15 cm in length, 1.14 cm diameter), ≈1 × 10^–5^ Torr, 700 °C, 3 h	red P (1 g), Sn (40 mg) SnI_4_ (20 mg)	BP	123
CVT‐low pressure	quartz ampoule (12 cm long), 873 K, 24 h	red P (500 mg), AuSn (364 mg) and SnI_4_ (10 mg)	BP	121
CVT‐low pressure	evacuated Pyrex tube, 923 K, 5 h	red P (500 mg), Sn (20 mg), SnI_4_ (10 mg)	BP	114
Mechanical milling	10 stainless steel balls (10 mm or 12.7 mm in diameter), Ar, 1 h	red P	BP, BP‐AB composite	115
Mechanical milling	7 g, 9, 20 mm; 20, 10 mm; 30, 6 mm in dia, Ar (1.2 MPa), 400 rpm, 12 h	red P	BP	116

### 3.1 High‐Pressure Route

**Figure 4:**
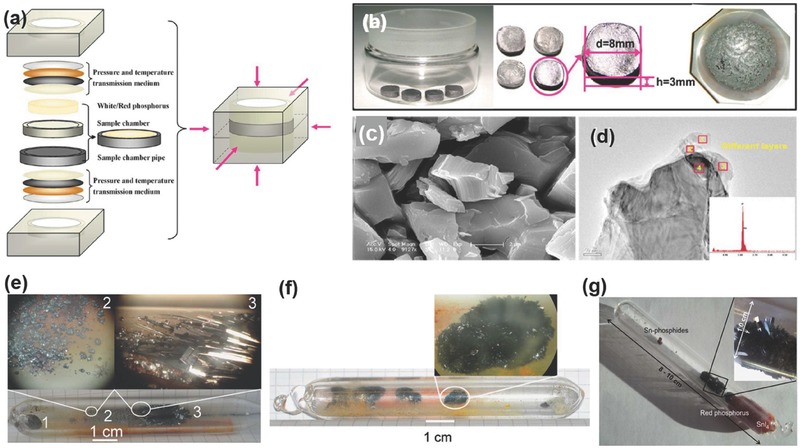
Bulk BP synthesis: a) Schematic of HTHP experimental setup. b) Photograph of the WBP sample. c,d) SEM and TEM images of WBP. Reproduced with permission.^107^ Copyright 2012, American Chemical Society. Photograph of silica ampoule after the CVT reaction at a temperature of e) 873 K and f) 923 K where 1, 2 and 3 represent the bulk residue, violet phosphorus and the main product black phosphorus, respectively. Reproduced with permission.^112^ Copyright 2008, Elsevier. g) Silica glass ampoule after CVT synthesis showing large bunches of BP. Reproduced with permission.^122^ Copyright 2014, Elsevier.

Bridgman^103^, ^104^ explained the discovery of black phosphorus as an event that occurred when ordinary white phosphorus (white P) was forced to change into red phosphorus (red P) under high hydrostatic pressure. The transition from white to black phosphorus occurred when pressure (≈11 000 to 13 000 kg cm^–2^) was applied at room temperature to the white phosphorus through a kerosene medium at 200 °C, in an oil bath controlled by a thermostat. Black phosphorus exhibits two distinct characteristic fractures: it is coarsely granular, like sugar (not in its crystalline form), and it is fibrous with a metallic luster (similar in appearance to graphite). Bulk BP was also produced under a constant pressure of 10 kbar by heating red phosphorus to 1000 °C and slow cooling it to 600 °C at a cooling rate of 100 °C h^–1^.^35^, ^105^, ^108^ The high‐pressure environment was provided by a cubic‐anvil type of apparatus. Synthesized BP should be kept in an inert atmosphere. A high‐temperature high‐pressure (HTHP) method (see **Figure** **4**a) was reported for the preparation of BP using a cubic‐anvil high‐pressure apparatus under a pressure of 2 to 5 GPa, where the blocks of white P and red P powder were shaped into cylindrical capsules (3‐mm thick and 10‐mm in diameter) in a chamber made of sintered boron nitride.^107^, ^117^, ^118^ Subsequently, pressure was applied with six tungsten carbide anvils to the cube containing the sample and a heater. The successfully synthesized WBP (black P obtained from white P) under different conditions has a metallic luster and a dark gray color. Figure 4 depicts scanning electron microscopy (SEM) and transmission electron microscopy (TEM) images of WBP. It was revealed that the synthesized WBP could be easily distinguished from their appearance (metallic luster, dark grey in color). High resolution TEM (HRTEM) confirmed the puckered layer structure with polycrystallinity as seen from the concentric diffraction rings and irregular diffraction spots.

### 3.2 Recrystallization From Bi Flux

The preparation of needle‐shaped BP single crystals from a solution of white P in liquid bismuth, usually called the bismuth‐flux method, was reported by Brown and Rundqvist^59^ in 1965. Maruyama et al.^60^, ^119^ adopted the same method to obtain BP from a solution of white P or polycrystalline BP in Bi. From the reports of Iwasaki et al.,^120^ the needle‐shaped BP crystals grown by the Bi‐flux method demonstrate a 2D Anderson localization in electrical properties at low temperatures; this has not been reported in crystals obtained from high‐pressure and high‐temperature routes. It confirms the significant dependence of the BP electrical conductivity at low temperatures on the growth method. Baba et al.^109^ reported on BP crystals grown in different shapes from a solution of white P in liquid Bi via an improved Bi‐flux method, with reduced chemical impurities. As red P does not dissolve directly in Bi, it cannot be used as the starting material in the Bi‐flux method. It is also very difficult to have highly pure white P due to its chemical activity.^109^ On the other hand, white P can be readily converted by the action of heat or light on red P. Though the commercial white P can be purified by water‐steam distillation, it is very challenging to remove the sulfur, selenium and arsenic impurities. The entire process of converting the red P to white P and the crystallization of BP was carried out in an evacuated quartz‐glass apparatus inside vacuum because white P should not be exposed to air as it is poisonous, highly reactive and inflammable in air.^109^ A quartz ampoule containing the white P (with a melting point of 44.1 °C) was melted at 80 °C and then moved towards the Bi (heated to 300 °C). Later, the ampoule was placed in an electric furnace, heated to 400 °C for 48 h and finally cooled down.

### 3.3 Chemical Vapor Transport

Single crystals of BP can be grown by the chemical vapor transport (CVT) method.^110^, ^111^, ^121^ The process is detailed as follows: red phosphorus (500 mg), Gold Tin metal (AuSn) (364 mg) and Tin (IV) Iodide (SnI_4_) (10 mg) were sealed in an evacuated 12‐cm‐long quartz ampoule. The charged end of the ampoule was placed horizontally at the center of a single‐zone tube furnace. The ampoule was slow heated to 873 K for 10 h and maintained at the same temperature for 24 h. The ampoule was subsequently cooled to 773 K at a rate of 40 K h^–1^. BP single crystals larger than 1 cm were crystallized in the form of flakes at the cold end of the ampoule. The low‐pressure route, with the use of a mineralizer as the reaction promoter, was of interest because of its high yield in non‐toxic experimental conditions.^112^, ^113^, ^114^, ^121^, ^122^, ^123^ The red phosphorus, converted to BP by SnI_4_ mineralization, gives red phosphorus and Au as its byproducts.^113^, ^124^

Nilges et al.^112^ used the mineralizer SnI_4_ prepared from tin powder (1.2 g) and iodine (4 g) in 25 mL toluene (starting materials) that was refluxed for 30 min; the synthesis process adopted by them for the low‐pressure route is subsequently described. AuSn was synthesized from an equimolar mixture of gold and tin in a sealed evacuated silica ampoule, and AuSn was adopted as a binary precursor to accelerate the reaction of polyphosphide Au_3_SnP_7_ at elevated temperatures prior to the transport reaction. The starting materials were melted by a H_2_/O_2_ burner before the growth process. The starting materials of red phosphorus, AuSn and SnI_4_ were placed in the silica ampoule (10 cm in length, 10 mm in diameter), which was evacuated to a 10^–3^ mbar pressure and placed in a muffle furnace (873 K, 23 h), resulting in the formation of BP crystals (>1 cm). Nilges et al. also reported a total conversion of red P to BP by extending the reaction time to 70 h at 923 K. The formation of violet phosphorus was an intermediate step in the transformation of red to black phosphorus and therefore, a reaction time longer than 32.5 h or a reaction temperature of 923 K promotes the complete conversion of violet to black phosphorus.^112^ The Sn to SnI_4_ ratio is the most critical factor for the successful growth of high quality BP bulk crystals.^123^ Figure 4e,f shows the photograph of the silica ampoule after the reaction at temperatures of 873 K and 923 K, with a clear identification of the bulk residue, the violet phosphorus and the main product (black phosphorus). The final BP product was collected and washed repeatedly with hot toluene and acetone for an enhanced removal of the residual mineralizer.^114^ A modified mineralizer‐assisted short‐way transport reaction involving red phosphorus, Sn/SnI_4_ as the mineralization additive to promote short reaction times, and high‐quality large BP crystals was also reported in the literature.^122^ Moreover, the silica glass ampoule (10 cm length, 1.0 cm diameter and a wall thickness of 0.25 cm) containing Sn (20 mg), SnI_4_ (10 mg) and red phosphorus (500 mg) was evacuated and placed horizontally in a muffle furnace, set to a temperature of 650 °C and then cooled for 7.5 h to 550 °C. A clear picture of the silica glass ampoule after the synthesis, where SnI_4_ (orange) and red phosphorus (red) from the gas phase are condensed at the right hand side of the ampoule, is shown in Figure 4g. Additionally, Sn‐Phosphides can also be observed as small round spheres resulting from the excessive Sn reaction.

### 3.4 Mechanical Milling

BP can be prepared by a mechanical milling process using a mixer mill and a planetary ball‐mill apparatus with red phosphorus powder as the starting material.^115^ The process was carried out in a stainless steel pot with 10 stainless steel balls (10 mm or 12.7 mm in diameter) in Ar atmosphere for 1 h. Composites with a composition of BP and acetylene black (AB) (80 wt% BP, 20 wt% AB) were prepared by a similar milling technique (a mixer mill) for 1 h. The mixer mill apparatus was found to yield black phosphorus with higher crystallinity depending on the difference in the impact of the mechanochemical reaction for two types of ball‐mill apparatuses.^115^, ^125^ Additionally, the mixer mill apparatus provides a more efficient impact interaction than the planetary ball‐mill apparatus in the process of converting red phosphorus to black phosphorus. The composites (BP‐AB) showed less agglomeration with secondary particles (1–5 µm) compared to the synthesized BP, which showed more agglomeration and formed secondary 30 µm‐sized particles. The mechanical milling of BP and AB leads to a decrease in the size of the secondary particles of the composites. Sun et al.^116^ synthesized BP from red phosphorus by means of a high‐energy mechanical milling method in a ball‐mill instrument. Red phosphorus (7 g) was cleaned with 5% sodium hydroxide solution and distilled water for the removal of oxides. A stainless steel vessel (with a 0.1 L capacity) containing red phosphorus and different sized stainless steel balls (9, 20 mm; 20, 10 mm; 30, 6 mm in diameter) was sealed in an Ar‐filled (1.2 MPa) glove box and rotated for 12 h at 400 rpm.

## 4 Fabrication of Few‐Layered BP

The higher intralayer strength and weaker interlayer cohesion of phosphorene enables their top‐down synthesis by the cleaving of layers from bulk BP.^126^ Apart from the dry and wet transfer methods, few‐layered BP can also be fabricated by other methods and we will discuss them in detail in this section.

### 4.1 Dry Transfer Methods: Mechanical Exfoliation

The mechanical exfoliation (ME) technique is widely adopted in the fabrication of BP.^105^, ^121^, ^127^, ^128^, ^129^ Because of its simplicity and ability to produce high‐quality materials, this technique was first utilized in the fabrication of graphene, and it is also growing to be more attractive for the synthesis of other 2D materials (much earlier for MoS_2_) that are different from their bulk forms.^130^, ^131^, ^132^, ^133^, ^134^ According to theoretical studies, 2D materials may be intrinsically unstable after exfoliation, which was later explained by the fact that the exfoliated monolayers are stabilized by the formation of ripples, enabling the extension of 2D materials to the third dimension.^135^, ^136^, ^137^ The process involved in this method is difficult for obtaining uniform samples, as one can obtain flakes that have different types of layers; the ME process is also a time‐consuming one.^138^

The main challenge of ME is that the performance of BP‐based devices not only depends on the number of layers but also on the quality of the crystal lattice.^35^, ^86^, ^105^ Moreover, the top‐down approach of mechanical exfoliation has been of significance in obtaining the highest‐quality samples. Because thickness is one of the critical parameters that defines the electronic, optical, and thermal properties of two‐dimensional crystals, it is natural to ask if we can achieve monolayer phosphorene. The method seems to be neither high throughput nor high yield.^139^ The studies of BP using mechanical exfoliation method is tabulated in **Table** **2**. A single sheet can be exfoliated when the van der Waals attraction present between the first and second layers can be overcome without destroying the consecutive sheets. The first identification of the exfoliated flakes is usually achieved by optical contrast in a microscope, where the regions with different colors represent phosphorene flakes of different thicknesses.^63^

**Table 2:** Mechanical Exfoliation

Method	BP Thickness (nm)	Substrate	Substrate thickness (nm)	Device structure	Protection	Reference
ME	0.7 (≈1 L), 1.1 to 1.6	SiO_2_/Si	285/90 (SiO_2_)	–	–	57
ME	1.3 (≈2L)	SiO_2_/Si	275 (SiO_2_)	–	–	63
ME	≈200–20	glass	–	–	PDMS	105
ME	20, <5	SiO_2_/Si	300 (SiO_2_)	–	Resist (ZEP520A)	127
ME	≈10–12	SiO_2_/Si	300 (SiO_2_)	–	PMMA	128
ME	25	SiO_2_	285 (SiO_2_)	–	–	121
ME	2L, 3L, 4L	SiO_2_/Si	275 (SiO_2_)	–	–	152
ME	1L, 2L to 5L	SiO_2_/Si	275 (SiO_2_)	–	–	154
ME	≈25	–	–	–	PDMS	108
ME‐Scotch‐PDMS	1.6 (≈2L)	SiO_2_/Si	285 (SiO_2_)	–	PDMS	156
ME‐Scotch‐PDMS	8–30	SiO_2_/Si	285 (SiO_2_)	–	PDMS	155
ME‐Scotch‐PDMS	2.8 (≈2L)	SiO_2_/Si	305/291 (SiO_2_)	–	PDMS	150
ME‐Scotch‐PDMS	6 to 47 (≈11L to 90L)	SiO_2_/Si	300 (SiO_2_)	–	PMMA/MMA	28
ME‐Scotch	>15	SiO_2_/Si	300 (SiO_2_)	–	AlO_x_	36
ME‐Scotch	10, 8, 5	SiO_2_/Si	90 (SiO_2_)	–	–	35
ME‐Scotch	9.5–29.6	SiN (free standing)	200	–	PMMA/PVA	148
ME‐Scotch	12	SiO_2_/Si, glass	285 (SiO_2_)	–	PDMS	37
ME‐Scotch	≈10	SiO_2_/Si	300 (SiO_2_)	–	PMMA	22
ME‐Scotch	2.8 (≈5L)	SiO_2_/Si	300 (SiO_2_)	–	–	54
ME‐Scotch	10L, 15L, 25L	–	–	–	–	2
ME‐Scotch	4.8	SiO_2_/Si	300 (SiO_2_)	FET sensor	–	29
ME‐Scotch	1L to 6L	SiO_2_/Si	275 (SiO_2_)	–	–	129

#### 4.1.1 First‐Principles Calculations on Mechanical Exfoliation

An important question arises when we think of how the origin of the research interest in few‐layered BP occurred a very long time (almost a century) after its bulk synthesis.^103^, ^140^ Thanks to the mechanical exfoliation techniques, the realization of phosphorene from its bulk counterpart is made possible. There is almost no report available to supplement a solid understanding of the exfoliating mechanism for advanced practical applications. Mu and Si^55^ described the sliding processes of bilayer phosphorene by calculating the sliding energies (*E*_s_) using first‐principles calculations with density functional theory (DFT) SIESTA code, including the van der Waals (vdW) correction to address the above‐mentioned issue. Bilayer phosphorene is favored to AB stacking due to its minimum *E*_s_; the energy curve generated is also reasonable when the vdW interaction is taken into account.^141^ To be more specific, all the possible sliding pathways are explicitly depicted in this energy surface and it will serve as an important guide for experimental research.

Therefore, the exfoliation of the bilayer phosphorene to create a monolayer along the *x*‐direction requires overcoming the maximum energy barrier of approximately 270 meV. In the case of the sliding process along the *y*‐direction, the energy barrier is approximately 110 meV, which is two times smaller in magnitude than that of the *x*‐direction. The important fact is that the minimum energy barrier exists when the sliding is along the diagonal (*xy*) direction as a result of the low energy barrier of approximately 60 meV that originates from the puckered structure of BP; this energy barrier is slightly larger than that of bilayer boron nitride (h‐BN), graphene and other lubricant materials.^142^, ^143^, ^144^ To reduce the energy barrier in phosphorus allotropes, the blue phosphorene might be a good candidate with its more planar in‐plane configuration.^145^, ^146^ The comparable energy barriers of MoS_2_, from the experimental and theoretical perspectives, can also be found in literature.^147^ The significance of this is that the interlayer sliding constraints determine the contribution of interlayer Coulomb interactions to the sliding energy profile which in turn results in different sliding pathways. Hence, the optimal pathway is to slide the BP along the diagonal direction.

#### 4.1.2 A Scotch‐Tape‐Based Mechanical Exfoliation Method

Mechanical exfoliation using scotch‐tape has been reported in many studies.^28^, ^29^, ^35^, ^36^, ^37^, ^148^ The isolation of single‐layer phosphorene that can be performed by means of a classical scotch tape (also called as blue Nitto tape)‐based mechanical exfoliation is divided into two steps: (i) Exfoliation of BP layers from bulk BP using scotch tape and (ii) transfer of the exfoliated BP layers onto the substrate (usually SiO_2_). The transfer is performed by aligning the desired BP flake to the targeted substrate. The exfoliation process must be carried out inside a glove box and kept under vacuum. It is then possible to explore the enormous quantities of information obtained to develop a suitable means for the protection of few‐layer BP from degradation. Other preventive measures should also be emphasized due to the easy degradation of the material when exposed to air and the challenges faced in protecting the surface from oxidation.

**Figure 5:**
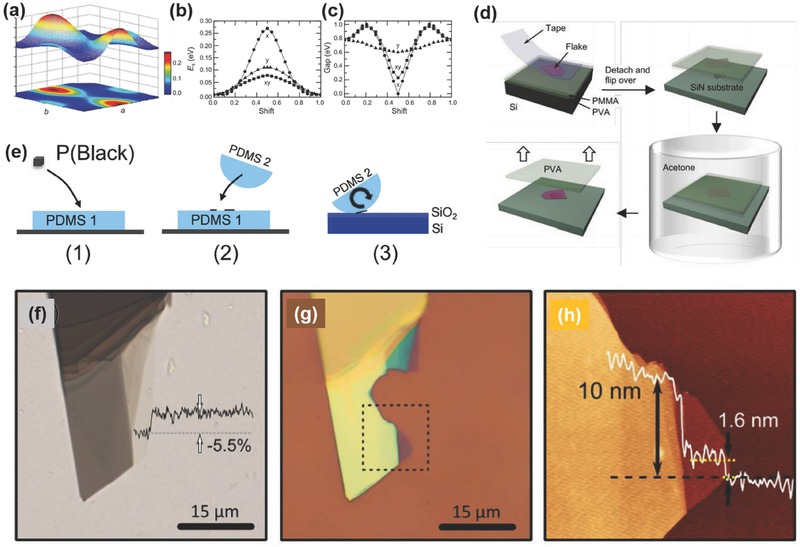
Mechanical Exfoliation of BP: The bilayer BP's a) sliding energy surface, b) sliding energy profiles, and c) band gaps of the three pathways. Symbols x, y and xy label the pathways along the x, y and diagonal directions, respectively. Reproduced with permission.^55^ Copyright 2015, Institute of Physics. d) Steps involved in the flake preparation and transfer process. Reproduced with permission.^148^ Copyright 2015, Nature Publishing Group. e) Three‐step exfoliation of BP with PDMS. 1) Exfoliation on PDMS‐1, 2) flakes are rolled on the semi‐spherical PDMS‐2 stamp and 3) the stamp is rolled on the SiO_2_/Si substrate. Reproduced with permission.^150^ Copyright 2015, Nature Publishing Group. f–h) Isolation of few‐layer BP. f) Transmission mode optical microscopy image of few‐layer BP on the PDMS substrate. Optical transmittance line profile to highlight the reduction of approximately 5.5% in the thinner part of the flake. g) Bright‐field optical image of the same flake after transferring to the SiO_2_/Si substrate (flake was broken during the transfer). h) AFM image of the dashed square region in (g) with a topographic line profile taken along the horizontal dashed black line. Reproduced with permission.^156^ Copyright 2014, IOP.

**Figure 6:**
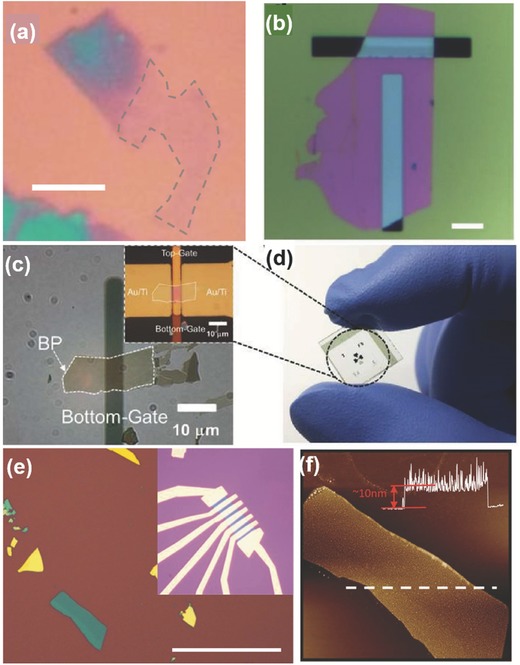
a) Optical image of mechanically exfoliated phosphorene flake (scale bar: 6 µm). Inset: AFM line scan showing a height of 0.7 nm and the monolayer region marked by a grey dashed line. Reproduced with permission.^240^ Copyright 2015, Nature Publishing Group. b) optical image of the PMMA/PVA‐assisted mechanically exfoliated BP flake suspended on slits (scale bar: 15 µm). Reproduced with permission.^148^ Copyright 2015, Nature Publishign Group. c) Optical micrograph of BP nanosheet on the Al_2_O_3_/patterned bottom gate. Inset: optical image of the fabricated dual‐gate BP FET. d) Photograph of dual‐gate device fabricated on glass. Reproduced with permission.^37^ Copyright 2015, American Chemical Society. e) Optical image of the BP flake on Si/SiO_2_. Inset: Same flake after fabrication of electrical contacts (scale bar: 50 µm). f) AFM image. Reproduced with permission.^22^ Copyright 2014, American Institute of Physics.

Researchers have adopted different approaches to protect BP from oxidation once it is mechanically exfoliated. Saito et al.^127^ reported that they covered the BP flakes with a resist (ZEP 520 A) immediately after exfoliation. The resist on the substrate was eventually removed by placing the substrate in N‐methyl‐2‐pyrrolidone (NMP) for 40–60 min at 323 K, followed by sprinkling acetone and drowning it in isopropyl alcohol. Luo et al.^148^ used 1‐µm‐thick poly(vinyl alcohol) (PVA) baked at 70 °C for 5 min, coated the PVA with a 200‐nm‐thick poly(methyl methacrylate) (PMMA) and baked the resulting stack at 70 °C for 5 min. The exfoliated BP flakes were transferred to the PMMA/PVA stack which was then cleaved off and flipped over to be mounted on a glass plate for further investigation. The flake, together with the PMMA/PVA stack, was transferred to the desired substrate (200‐nm‐thick free standing SiN). The sample was drenched (for >12 h) in acetone (>70 mL) to remove the PMMA/PVA and then dried with nitrogen (see **Figure** **5**d). They also suggested that using a large amount of acetone along with a long soaking time is needed for the effective removal of PMMA. No baking or annealing was performed through the entire processing steps to prevent excessive oxidation and also to retain the BP crystallinity. An optical image of the exfoliated BP flake suspended on slits is shown in **Figure** **6**b. The degradation of BP was also found to be minimized by coating with only PMMA.^36^, ^105^, ^128^ The approximate time to cover the flake with PMMA after exfoliation (investigation under a microscope) was estimated to be less than 30 min.^22^ An optical image and AFM of the BP flake on Si/SiO_2_ and the device fabricated by Koenig et al.^22^ is shown in Figure 6e,f. Another effective approach is to adopt atomic‐layer‐deposited AlO_x_ overlayers to effectively suppress the ambient degradation of BP.^36^

BP nanosheets were mechanically exfoliated from single crystal bulk BP using Scotch tape and PDMS elastomer on 285‐nm‐thick SiO_2_/p^+^‐doped silicon and glass substrates.^37^ Figure 6c,d presents the optical micrograph of the BP nanosheet on an Al_2_O_3_/patterned bottom gate and also the photograph of a fabricated dual‐gate BP FET device on glass. Wang et al.^105^ exfoliated BP flakes onto a PDMS stamp on a glass slide. The glass slide is kept in a vacuum chamber (p ≈ 5 mTorr) immediately after the careful identification of promising flakes for further usage by optical microscopy. The pre‐patterned substrate that fits well with the geometry of the chosen flake was used. Wang et al. carried out the transfer by aligning the selected BP flake to the target device area on the substrate. The slide was then lowered to make contact between the PDMS and the substrate. The PDMS was later peeled carefully, leaving BP on the substrate because of the van der Waals forces existing between BP and the surface. This efficient dry transfer method was ≈70% successful in the fabrication of good‐quality suspended BP nanoelectromechanical systems (NEMS) with sophisticated structures. They were also able to preserve the crystal quality better than with the conventional lithography process accompanied by wet transfer techniques. These wet transfer techniques involve the exposure of BP flakes to wet chemical processes, causing undesired chemical reactions and prolonged time in the ambient condition that leads to unwanted oxidation.

**Figure 7:**
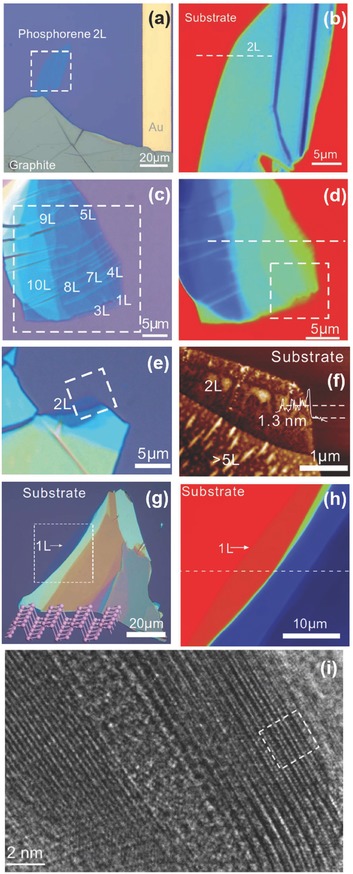
a) Optical micrograph of the device fabricated by the bi‐layer BP (labeled as “2L”). b) PSI image of the region marked as a square with a dashed line in (a). c,d) Optical image and PSI image of another exfoliated few‐layered BP flake, respectively. Reproduced with permission.^152^ Copyright 2015, American Chemical Society. e) Optical image of 2L BP. f) AFM image of 2L BP taken from the region indicated by the box with a dashed line in (e). Reproduced with permission.^63^ Copyright 2014, American Chemical Society. g) Optical micrograph of monolayer phosphorene (labeled as “1L”). Inset: Molecular structure representation of phosphorene. h) PSI image of the dashed‐line square given in (g). Reproduced with permission.^154^ Copyright 2015, Nature Publishing Group. i) HRTEM image of a 2D‐phosphane layer. Reproduced with permission.^54^ Copyright 2014, the authors.

Favron et al.^54^ performed the exfoliation of BP in the dark or inside a nitrogen‐filled glove box to protect it from degradation. A SiO_2_/Si substrate was coated with 20 nm of parylene C to minimize the influence of hydrophilicity on the surface. The presence of parylene is used for a clear identification of the exfoliated flakes and also as a protection against degradation.^28^, ^149^, ^150^ The samples can also be washed with acetone, methanol and isopropanol (approximately 1 minute for each step) so that the residue from the scotch tape can be removed; this is followed by baking at 180 °C for 5 min to remove the remaining solvent.^57^ An optical image of a mechanically exfoliated monolayer BP flake, as reported by Wang et al.,^57^ is shown in Figure 6a. Liu et al.^151^ spin‐coated PMMA onto exfoliated BP flakes (twice, at 2000 rpm, for 1 min each time) and anisole solvent was later baked out at ≈150 °C for 5 min. The PMMA/BP/SiO_2_/Si stack was soaked in 2 m KOH to etch the SiO_2_/Si and release the PMMA/BP. The KOH will not significantly affect BP because BP is more stable than red phosphorus. The PMMA/BP films were rinsed in ultrapure deionized water to remove the etching residues, and the remaining water was removed from the interface of the PMMA/BP films by successive ≈70 °C and ≈150 °C heating steps, with each step performed for 10 min. Later, the PMMA was dissolved with hot acetone vapor (at ≈45 to 55 °C) in necked Erlenmeyer flasks. The grids were stored in a N_2_ glove box, prior to characterization, to prevent BP degradation in ambient conditions. The overall process can be classified in the following steps: (1) exfoliate BP onto the SiO_2_/Si wafer; (2) spin coat PMMA onto the sample; (3) etch the SiO_2_/Si with aqueous 2 m KOH solution; (4) rinse the PMMA/BP sample in an H_2_O bath and (5) transfer the BP flakes onto another SiO_2_/Si wafer. To slow the reaction of phosphorene with moisture and other possible reactants from the environment, Zhang et al.^63^, ^152^ placed the exfoliated samples in a microscope‐compatible chamber with a slow flow of nitrogen as the protecting gas. **Figure** **7** represents the morphological studies of the bi‐layer BP and the few‐layered BP flake grown by mechanical exfoliation.^63^, ^152^

Atomic force microscopy (AFM) and Raman spectroscopy have been employed to study the sample thickness of TMDs but they are not reliable for studying one or two layers of phosphorene.^153^ Because the scanning rate of AFM is slow in comparison to the fast degradation of phosphorene in ambient conditions, AFM may show an error of one or two layers due to the large surface roughness. There is also the possibility of introducing potential contaminants from the AFM system. Interestingly, phosphorene has a non‐monotonic dependence on the layer number ascribed to the complicated Davydov‐related effects, unlike TMDs, which have a monotonic dependence in the Raman mode frequency.^54^ The phosphorene can also be damaged by the high‐power laser adopted in characterization by Raman spectroscopy. Yang et al.^154^ reported a different approach for determining the layer number by means of optical interferometry i.e., phase‐shifting interferometry (PSI) to measure the optical path length (OPL) reflected from the exfoliated phosphorene by analyzing the digitized interference pattern. The virtual thickness of a phosphorene flake is amplified by more than 20 times in the optical interferometer due to the multiple interfacial light reflections, and this is helpful for easy identification of the flakes. PSI adopts non‐focused and very low‐density light from a light‐emitting diode source to achieve fast imaging without causing any damage to the phosphorene samples. Figure 7g,h shows an optical micrograph of monolayer phosphorene and its PSI image. HRTEM can be employed for structural analysis of few‐layered BP. The puckered structure of 2D‐phosphane is clearly revealed from the HRTEM image (see Figure 7i) reported by Favron et al.^54^

#### 4.1.3 Modified Mechanical Exfoliation Technique

Modified mechanical exfoliation was introduced by Castellanos‐Gomez et al.^155^, ^156^, ^157^ to optimize the deposition of atomically thin BP flakes. Conventional mechanical exfoliation with adhesive tape yields a low density of few‐layer BP flakes, leaving traces of adhesive on the surface and thereby reducing the quality. A better solution could be using an intermediate viscoelastic surface in exfoliation, which increases the yield and further reduces the contamination of exfoliated flakes. Commercially available bulk BP was cleaved multiple times by blue Nitto tape. Later, the tape containing thin BP crystallites was slightly pressed against a poly‐dimethylsiloxane (PDMS)‐based substrate and peeled off rapidly. Finally, the thin flakes present on the surface of the PDMS substrate were moved to the desired substrates by simply putting the PDMS substrate in gentle contact with the new acceptor substrate and peeling it off slowly (approximately 5–10 minutes to peel off the stamp completely from the surface). Island et al.^155^ reported that long‐term exposure to ambient conditions results in a layer‐by‐layer etching process of BP flakes, which they were able to etch down to a single‐layer (phosphorene) thickness (≈0.7 nm). Their results demonstrated that the initial exposure of BP flakes to air results in n‐type doping, whereas a longer exposure to air leads to strong p‐type doping ascribed to the presence of absorbed water.

Another modified Scotch‐tape exfoliation technique was reported in the literature where BP was first exfoliated onto a flat PDMS stamp and then transferred onto a curved PDMS stamp.^28^, ^150^, ^158^ Later, the curved PDMS stamp covered with flakes is rolled onto the desired substrate. BP films were also produced by the micromechanical cleavage of bulk BP crystals directly onto a PDMS stamp.^108^ As a result of the viscoelastic properties of PDMS, the BP film adheres to the fiber end when the film is gently lifted up from the PDMS stamp.^156^ Tayari et al.^28^ deposited 300 nm of copolymer (methyl methacrylate, MMA) and 200 nm of polymer (polymethyl methacrylate) and annealed the deposition at 170 °C for 15 minutes to protect BP field‐effect transistors (FETs) against degradation. Figure 5f–h shows a transmission mode optical microscopy image of few‐layer BP on a PDMS substrate, a bright field optical image of the same flake after transferring to a SiO_2_/Si substrate and an AFM image. The significant role of the polymer layer is to form a water‐impermeable superstrate that suppresses oxidation and hence, the BP surface is free of the surface roughening caused by oxidation.^22^, ^28^, ^36^, ^54^, ^156^

### 4.2 Wet Transfer Methods: Liquid‐Phase Exfoliation

**Figure 8:**
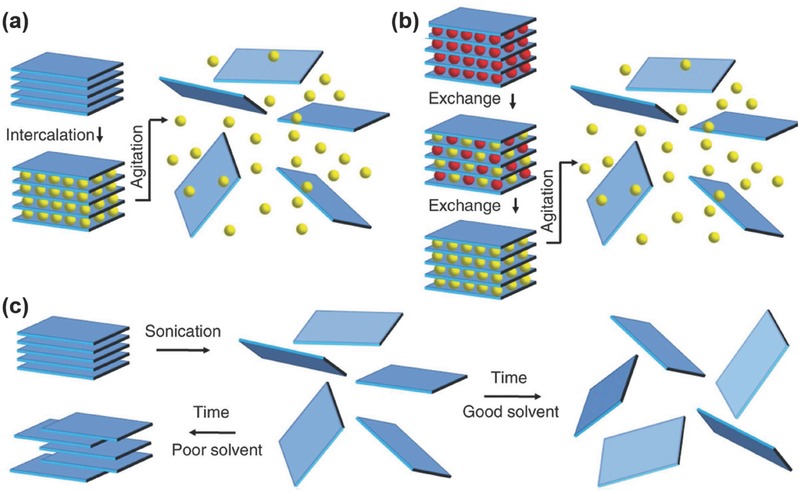
Schematic illustration of liquid exfoliation mechanism. a) Ion intercalation: Ions (yellow spheres) are intercalated between the layers in the liquid, swelling the crystal and weakening the interlayer attraction. Shear, ultrasonication or thermal agitation can completely separate the layers, thus producing the final exfoliated dispersion. b) Ion exchange: Ions found between the layers in layered materials balance the surface charge on the layers. These ions (red spheres) can be interchanged in the liquid for other larger ions (yellow spheres), and further agitation yields the exfoliated dispersion. c) Sonication assisted exfoliation: The layered material is sonicated in a solvent to form a nanosheet by means of exfoliation. In the case of a good solvent with an appropriate surface energy, stabilized nanosheets are formed; bad solvents cause reaggregation leading to sedimentation. Reproduced with permission.^135^ Copyright 2013, AAAS.

Liquid exfoliation or liquid phase exfoliation (LPE) can be broadly classified into the following basic categories of (i) oxidation followed by subsequent dispersion into suitable solvents, (ii) ion intercalation, (iii) ion exchange, (iv) ultrasonication‐assisted exfoliation and (v) shear exfoliation.^135^ **Figure** **8** describes the detailed schematics of the liquid exfoliation mechanisms. In the LPE method, one can achieve large quantities of dispersed nanosheets of layered materials which are more suitable for industrial‐scale applications. The liquid exfoliation of layered compounds in modern research started and progressed based on the graphite intercalation compounds and graphite oxide.^159^, ^160^ An earlier study reported in inorganic layered compounds showed the exfoliation of vermiculite clay in liquids via ion intercalation and shear mixing.^161^ A few studies were later demonstrated on the use of ultrasonic agitators for ion intercalation‐assisted exfoliation of TaS_2_, NbS_2_, MoS_2_ and layered oxides.^162^, ^163^, ^164^, ^165^ The LPE method is selective to the dispersion liquid and hence, different solvent have different dispersibility and are found to have diverse nonlinear properties.^12^, ^166^ A solution‐based mechanical exfoliation process was also reported by mixing BP crystals with ethanol solution.^90^

The primary approach in liquid exfoliation is the oxidization first demonstrated in graphite.^159^ This is regarded as one of the oldest methods of exfoliating layered crystals with a low reducing potential followed by subsequent dispersion into suitable solvents.^135^ The amount and the type of oxides attached can be changed by means of oxidation to tune the properties such as electrical conductivity and luminescence.^167^ There are a few disadvantages with this method as it may be responsible for chemical groups and defects that scatter electrons and lead to high resistivity. Intercalation is a broadly adopted method in liquid exfoliation as the layered materials can adsorb guest molecules in the spacing existing between their layers, thereby forming inclusion complexes.^168^, ^169^ The intercalation process increases the layer spacing, weakens the interlayer adhesion and reduces the energy barrier to exfoliation.^135^ The role of the intercalants, such as n‐butyllithium^169^ and IBr,^168^ is to transfer charge to the layers and subsequently decrease the interlayer binding. The exfoliation process is completed with the aid of further thermal shock treatments^168^ or ultrasonication^169^ in a liquid. The final step is to stabilize the exfoliated flakes either by incorporating a surface charge or by adding surfactant.^168^ Although the intercalation techniques are sensitive to ambient conditions, they yield a large quantity of the exfoliated nanosheets.^169^, ^170^

Ion‐exchange methods make use of the exchangeable interlayer of cationic counterions present in clays and metal oxides.^161^, ^171^, ^172^ The most recent approach in liquid‐phase exfoliation was performed by ultrasonication in a solvent by exposing the 2D materials to ultrasonic waves that develop cavitation bubbles and collapse into high‐energy jets to cleave the layered crystallites, leading to exfoliation.^93^, ^135^, ^173^ It was demonstrated that the energy difference between the exfoliated and re‐aggregated flakes depends on the surface energy of the solvent and may be small if both the solvent and 2D material have the same surface energy.^160^ By ultrasonication with organic solvents and ionic liquids, shear exfoliation methods have been widely utilized in the liquid exfoliation of BP. Interestingly, there is a correlation between the cohesive energy of a 2D material and the solvent used that determines the capability of a particular solvent to achieve efficient exfoliation and create a stable dispersion.^174^ The cohesive energies can be evaluated by using two significant parameters: (a) the Hildebrand solubility parameter and (b) the Hansen solubility parameter. The Hildebrand solubility parameter is the measure of a solvent's total cohesive energy per unit volume and the Hansen solubility parameter is the measure of the dispersive, polar and hydrogen bonding components of a solvent's cohesive energy per unit volume.^175^, ^176^ If the Hansen solubility parameters of the desired 2D material and solvent are the same, that solvent would be the best candidate for exfoliation. **Table** **3** shows the experimental parameters and devices based on liquid exfoliation in previous literature.

**Table 3:** Liquid Exfoliation

Method	Parameters	Solvent	BP Thickness (nm)	Device structure	Centrifugation	Reference
Ultrasonication	750 W, 5 h, 2 g L^–1^	CHP	2	–	1 krpm, 180 min 2 krpm, 3 krpm, 4 krpm, 5 krpm, 10 krpm and 16 krpm for 2 h	191
Ultrasonication	400 W, 43 h, RT, ≈6 mg mL^–1^	ethanol	4 to 25	pulsed laser	4000 rpm, 60 min	93
Ultrasonication	1 mg mL^–1^, 2 h	IPA, NMP, EA	5 to 10	–	1500 rpm, 20 min	12
Ultrasonication	1 mg mL^–1^, 90 min	NMP	30 to 60	–	3000 rpm, 10 min	185
Ultrasonication	3 h	IPA	5	saturable absorber	1500 rpm, 20 min	239
Ultrasonication	4 h	NMP	3 to 9	saturable absorber mirror	1500 rpm, 45 min	95
Ultrasonication	3 h, 200 W, BP (5 mg), NMP (3 mL)	NMP	4.9 to 1.9	flexible memory device	7000 rpm, 20 min	118
Ultrasonication	40 kHz frequency, 80% power, 4 h	NMP	5.3, 2.8, 1.5 to 2.5	saturable absorber	3000 rpm, 10 min 12000 rpm, 20 min (5 to 12 L) 18000 rpm, 20 min (1 to 7 L)	67
Ultrasonication	130 W, 15 h, 10 µg mL^–1^	DMF, DMSO	5.8 to 11.8	–	2000 rpm, 30 min	182
Ultrasonication	15 mg, 20 mL, 12 h	DMF, DMSO	26	sensors	500 rpm, 30 min	195
Ultrasonication	40 kHz, 300 W, 10 h	NMP	≈0.6 to 2	saturable absorber	1500 rpm, 10 min	91
Ultrasonication	–	NMP	23	saturable absorber	–	85
Ultrasonication	20 mL, 0.1 mg mL^–1^, 10 h	NMP	0.84 to 4.22	sodium‐ion batteries	5000 rpm, 30 min	187
Ultrasonication	5 mg mL^–1^, 0.164 mol dm^–3^ of BP in NMP, 820 W, 37 kHz frequency, 30% power, 24 h, 30 °C	NMP	0.9 to 1.6, 3.5 to 5	–	1500 rpm, 45 min	186
Ultrasonication	10 mg, 20 mL, 0.5 mg mL^–1^	Different solvents	1 L, 2L	–	30 min	196
Ultrasonication	50 mg, 100 mL, 8 h	water	2	photosensitizers	1500 rpm for 10 min	114
Ultrasonication	BP, 0.4 g, 12.8 mmol, water (100 mL) 30 min, 20 kHz, 100 W	water	few nm to <20	biomedical	–	197
Ultrasonication (tip)	1 to 10 mg mL^–1^, 950 W, 30 to 300 min	water	9.4	lithium ion battery	1500 to 5000 rpm for 30 min	173
Ultrasonication (tip)	≈30 W power, ≈1 mg mL^–1^, 1 h	acetone, chloroform, hexane, ethanol, IPA, DMF, and NMP	16 to 128	–	500 to 15 000 rpm for 10 min	183
Ultrasonication (tip and bath)	BP (25 mg), NMP (25 mL), tip‐sonication (3 h, 1200 W, 19 to 25 kHz), bath‐sonication (300 W, 10 h, below 277 K)	NMP	≈1.5	photothermal agents	7000 rpm, 20 min, 12 000 rpm, 20 min	205
Ultrasonication, mechanical milling	up to 6 h, 30 min, 400 W, RT	DMF, NMP, DIGLYM, AN	2 to 5 (3 to 10 L), 20 to 30	gas sensing	5000 rpm, 10 000 rpm, 16 000 rpm	110
Ionic liquids	100 W, 24 h, ≈0.95 mg mL	I Ls, [HOEMIM] [TfO], [HOEMIM] [BF_4_]	3.58, 5.5, 8.9	–	4000 rpm, 45 min	200
Shear mixing	6 g of BP, 100 mL of NMP sonicated for 2 h, 700 mL of NMP shear mixed at 5000 rpm for 4 h	NMP	1 L, 2 L	–	–	196
Shear exfoliation	0.05 mg mL^–1^	NMP	≈0.9 to 3.5	rechargeable nanoscale battery	6000 rpm	207

#### 4.2.1 Theoretical Studies of Liquid Exfoliation of Phosphorene

The surface energies of the solvent and layered materials play a crucial role in tuning the exfoliation efficiency of the liquid‐exfoliation process. The surface energy of bilayer phosphorene was estimated to be ≈58.6 mJ m^–2^ based on a barrier energy of 60 meV, which fits well in the range of surface energies of solvents such as ethanol (22.0 mJ m^–2^), methanol (41.4 mJ m^–2^), water (72.7 mJ m^–2^) and formamide (57 mJ m^–2^).^55^ This theoretical study clearly supports the thinking that BP can be easily exfoliated by LPE.^55^, ^177^ Figure 5a–c shows the sliding energy surface, sliding energy profiles and band gaps of the three pathways of bilayer BP.

The quest for new exfoliating media is necessary to find environmental friendly solvents, thereby avoiding toxic and impractical solvents.^178^, ^179^, ^180^, ^181^ Therefore, Sresht et al.^126^ studied the LPE of phosphorene in the solvents dimethyl sulfoxide (DMSO), dimethylformamide (DMF), isopropyl alcohol (IPA), NMP and N‐cyclohexyl‐2‐pyrrolidone (CHP) using three molecular‐scale computer experiments to model the solvent–phosphorene interactions via atomistic force fields assisted by ab initio calculations and lattice dynamics. The energy needed to peel a single phosphorene monolayer from a stack of BP was measured, with a detailed explanation of the role of the wedges present in solvent molecules for initiating the exfoliation. The main findings of their simulations are given as follows: (1) the lower value of the primary minimum in CHP than the corresponding minimum for vacuum accounts for the easier aggregation of phosphorene monolayers in CHP than in vacuum; this indicates that CHP is a poor choice for LPE and agrees well with experimental results,^182^ (2) the cohesive strength of IPA is attributed to the hydrogen bonding networks between confined molecules,^182^ (3) the large energy barrier in DMF is due to (a) the small size and planarity of the DMF molecule giving rise to an enhanced intercalation ability and (b) the substantial cohesive energy that causes the high density, viscosity and boiling point, (4) DMSO, with its enhanced packing along with its high cohesive energy, high boiling point, density and viscosity, gives the optimal dispersion environment as supported by previous works,^182^, ^183^ and (5) the strong cohesive dipolar forces in certain solvents (DMSO, IPA) increase their suitability as stable dispersion media and therefore, solvent planarity improves the dispersion stability and leads to a greater degree of confinement of the solvent molecules between the 2D BP sheets.

The performance of a solvent based on the shape of its molecules in interfacial layers with phosphorene was clearly demonstrated by Sresht et al.^126^ In general, LPE starts by the penetration of a wedge of solvent molecules in the interlayer gap of 2D materials. Consequently, the solvents with planar molecules near the phosphorene surface (NMP, DMSO) act as molecular wedges for efficient intercalation.^126^ The intercalation can be successful if and only if the new phosphorene surfaces established by the solvent wedge are sustained by sorption forces that exist between the solvent molecules and the corresponding 2D material. Moreover, the cohesive energy density of the solvent molecules in the final interfacial layer of solvent, confined between the sheets, is high and continues to diminish if strong sorptive interactions exist between the confined molecules and the sheets, which favors the stable dispersion of phosphorene. The simulation studies showed that the phosphorene exfoliation is easier if the adhesion between the phosphorene and solvent is stronger than the cohesion between the solvent molecules.^126^ Accordingly, the solvent's molecular shape is more important and has to be taken into account in LPE.

#### 4.2.2 Ultrasonication‐Based Exfoliation

Different approaches were adopted in the ultrasonication of 2D materials including organic solvent‐based exfoliation, stabilizer‐based exfoliation, ionic liquid‐based exfoliation, salt‐assisted exfoliation, intercalant‐assisted exfoliation and ion exchange‐based exfoliation.^184^ Ultrasonication can be performed either by the bath sonication or the tip sonication technique. Bath sonication is used in majority of studies and hence, we do not mention it specifically and the general term of ultrasonication is used to denote “bath sonication” throughout this article.

##### 4.2.2.1 Organic‐Solvent‐Based Sonication

: One of the approaches used involved BP crystals mixed with ethanol solution and ultrasonicated (400 W) at room temperature (RT) for 43 h. The resulting solution was centrifuged (at 4000 rpm for 60 min) to eliminate larger particles and led to a final purified BP‐ethanol mixture (with a concentration of ≈6 mg mL^–1^).^93^ It was also reported that BP powder was obtained by grinding bulk BP crystal dispersed in different solvents such as IPA, NMP and ethyl alcohol (EA) to obtain 1 mg mL^–1^ dispersions, and the dispersions were ultrasonicated for 2 h. The dispersions were then allowed to settle for more than 24 h to enable the removal of large‐size sedimentations by centrifugation (at 1500 rpm for 20 min).^12^ The interesting fact behind the formation of thin BP nanoflakes in the solution is that the interlayer van der Waals bonding is broken down by the ultrasonic energy.^95^

**Figure 9:**
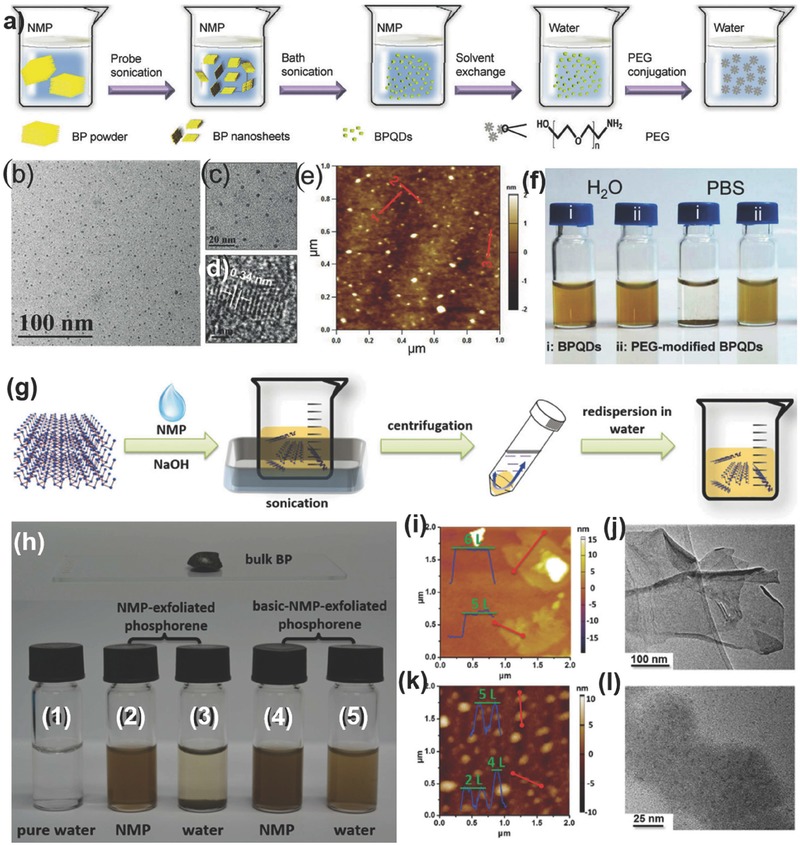
a–f) Synthesis and studies of the BP QDs: a) Steps followed in the fabrication and surface modification of BP QDs. b) TEM image. c) Magnified TEM image. d) HRTEM image. e) AFM image. f) Photo of synthesized BP QDs, i) PEG‐modified BP QDs and ii) BP QDs dispersed in water or phosphate‐buffered saline (PBS) solution. Reproduced with permission.^205^ g) Schematic of synthesis process of basic NMP‐exfoliated phosphorene. h) Picture of phosphorene dispersed in NMP and water. The five bottles contain the following: 1) pure water, 2) NMP‐exfoliated phosphorene in NMP, 3) NMP‐exfoliated phosphorene in water, 4) basic NMP‐exfoliated phosphorene in NMP, and 5) basic NMP‐exfoliated phosphorene in water. Height‐mode AFM images of i) 12000 and j) 18000 phosphorene, and TEM images of k) 12000 and l) 18000 phosphorene are shown. Reproduced with permission.^67^

The NMP solvent for obtaining stable, highly concentrated (≈0.4 mg mL^–1^) BP dispersions was used in LPE in most of the reported studies.^12^, ^67^, ^85^, ^91^, ^95^, ^118^, ^160^, ^166^, ^183^, ^185^, ^186^, ^187^ NMP and DMF are well suit for liquid exfoliation owing to their relatively high boiling points and surface tension (≈40 mJ m^–2^).^166^, ^183^, ^188^ However, phosphorene, extracted by NMP solution, cannot be used directly either in the fabrication of electronic devices or for optical investigations because of its poor volatility. The exfoliated nanosheets are further stabilized electrostatically or sterically by interaction with the solvent or by the presence of an adsorbed surfactant or a polymer.^160^, ^189^, ^190^ This method is more suitable for the large‐scale exfoliation of BP and a uniform dispersion can be achieved in the exfoliation medium. Specifically, the production yield is generally low and the phosphorene is unstable in other conventional solvents (water) limiting its applications.^67^ Guo et al.^67^ reported a basic NMP‐based LPE method for the fabrication of phosphorene with an excellent water stability, a controllable size, a number of layers and high yield. The schematic of the synthesis process of basic NMP‐exfoliated phosphorene is given in **Figure** **9**g. The basic NMP‐exfoliation process can be detailed as follows: bulk BP (15 mg) was added to a saturated NaOH NMP solution (30 mL) and sonicated for 4 hours at 40 kHz frequency and 80% power, and the phosphorene in NMP was separated and transferred to water by centrifugation (at 3000 rpm for 10 min) in order to remove the unexfoliated bulk BP. Figure 9h shows the photographs of phosphorene dispersed in NMP and water, with five bottles containing (1) pure water, (2) NMP‐exfoliated phosphorene in NMP, (3) NMP‐exfoliated phosphorene in water, (4) basic NMP‐exfoliated phosphorene in NMP, and (5) basic NMP‐exfoliated phosphorene in water. Compared to mechanical exfoliation, LPE is unable to yield 2D materials with an exact size and thickness. Guo et al.^67^ adopted different centrifugation speeds to control the phosphorene thickness. The supernatant solution was centrifuged at 12 000 rpm for 20 min to obtain 5.3 ± 2.0 nm thick (5 to 12 L) phosphorene samples with an average diameter of ≈670 nm (referred to as 12000 phosphorene) and further centrifuged at 18 000 rpm for 20 min to obtain 2.8 ± 1.5 nm thick (1 to 7 L) phosphorene samples with average diameter of ≈210 nm (referred to as 18000 phosphorene). The height‐mode AFM and TEM images of 12000 and 18000 phosphorene are shown in Figure 9i–l. The results are also demonstrated for the two conditions with and without the addition of NaOH to NMP, which confirms that a thorough exfoliation of BP was achieved by the basic NMP process (with NaOH) than by the NMP‐only exfoliation (without NaOH). Additionally, the phosphorene obtained from basic NMP‐exfoliation has excellent stability in both NMP and water. The basic NMP‐exfoliated phosphorene with a zeta potential of −30.9 mV is physically stable (in water) and has a more negative charge than the NMP‐exfoliated phosphorene with a zeta potential of −19.7 mV. The negative charge of phosphorene corresponds to the OH^–^ ions, obtained by adding NaOH, that are absorbed on the surface of phosphorene leading to an excellent stability in water.

**Figure 10:**
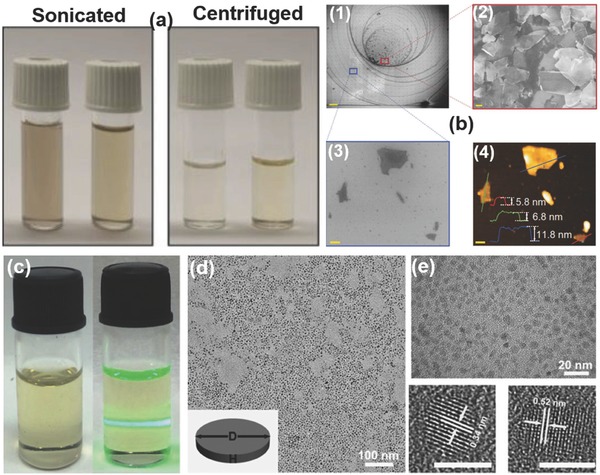
Liquid‐phase‐exfoliated BP nanoflakes: a) Photograph of few‐layered BP dispersions in DMSO and DMF solvents, (left) after sonication, and (right) after centrifugation and supernatant collection. b) Characterization of BP nanoflakes. 1) Low‐magnification SEM image of BP nanoflakes on the SiO_2_/Si substrate showing a “coffee‐ring” structure. Scale bar: 200 µm. 2) Magnified SEM image of the central area of the rings. Scale bar: 200 nm. 3) SEM image of individual BP nanoflakes present at the outer region of the coffee rings. Scale bar: 200 nm. 4) AFM image of the same area shown in (3) with height profiles (inset) corresponding to the drawn lines represented by the same colors. Reproduced with permission.^182^ c) Photographs of the BP QD suspension (left) and the Tyndall effect observed in the BP QD suspension (right). d) TEM image of BP QDs. e) Enlarged TEM image of BP QDs. HRTEM images of BP QDs with different lattice fringes. Scale bar: 5 nm. Reproduced with permission.^118^

**Figure 11:**
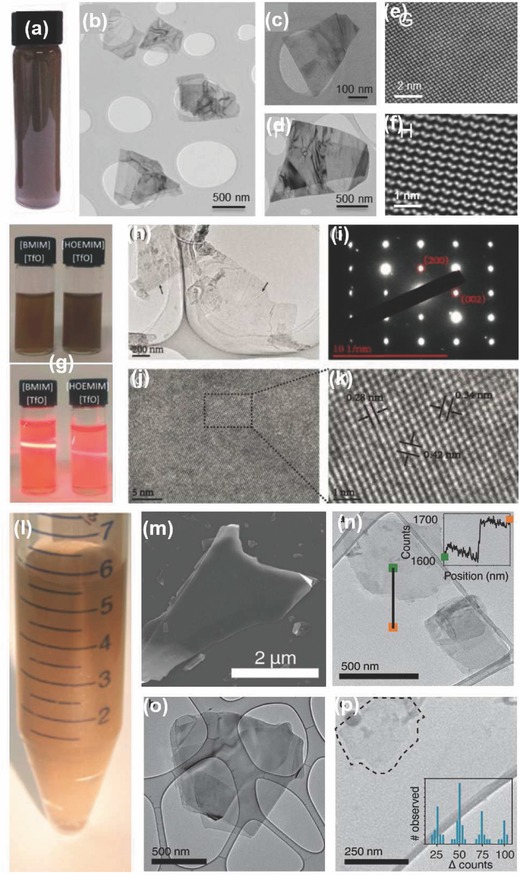
a–f) Liquid exfoliated FL BP: Dispersion in CHP. a) Photograph. b–d) Low‐resolution TEM images. e) Low‐by‐pass bright‐field STEM image. Dispersion in isopropanol. f) Butterworth‐filtered HAADF STEM image indicating the intact lattice Reproduced with permission.^241^ Copyright 2015, Nature Publishing Group. g–k) IL‐exfoliated BP nanosheets dispersions: g) Photograph of BP in [BMIM][TfO] and [HOEMIM][TfO] (top) and Tyndall effect of diluted dispersions (bottom). h–k) Electron microscopy studies of BP‐[HOEMIM][TfO]. h) Low‐magnification TEM image. The arrows points to wrinkles and distinguishable edges. i) SAED pattern. j) HRTEM image. k) Magnified HRTEM image of selected area in (j). Reproduced with permission.^200^ Copyright 2015, American Chemical Society. l–p) LPE exfoliated BP suspension in isopropanol: l) Photograph. m) SEM image. n–p) TEM images. p) TEM image of a monolayer BP. Inset (n): contrast change (ca. 75 counts) from a line profile across the 3L thick BP. Inset (p): Histogram of contrast changes from 100 flakes where the intensity change (25, 50, etc.) corresponds to monolayers, bilayers, etc. Reproduced with permission.^196^ Copyright 2015, American Chemical Society.

The improved basic NMP‐exfoliation method reported by Guo et al.^67^ may provide a better solution for this problem. Zhang et al.^118^ synthesized BP quantum dots (QDs), with an average size of 1.9 to 4.9 nm and excellent stability in NMP, using ice‐bath sonication (3 h, 200 W). The BP (5 mg) and NMP (1 mL) ingredients were grinded in a mortar for 20 min, transferred to a glass vial with 3 mL of NMP and sonicated for 3 h. The resultant dispersion was centrifuged for 20 min at 7000 rpm. All the experiments were conducted in a glove box except the sonication and centrifugation processes. Photographs of the BP QD suspension (left) and the Tyndall effect observed in the BP QD suspension (right) together with their TEM images are shown in **Figure** **10**c,d. Hanlon et al.^191^ reported on high‐quality, mono‐ and few‐layer BP (FL BP) created by LPE in the CHP solvent with remarkably stable nanosheets. Bulk BP is ground with a pestle and mortar immersed in CHP (with a concentration of 2 g L^–1^), which is sonicated for 5 hours to give a brown dispersion. At the time of sonication, 4 mL of the sample was collected for further sonication of ≈80 h, and it was subsequently centrifuged at 1 krpm for 180 min to yield a stable dispersion (designated as std‐BP). The stock dispersions were centrifuged at 1000 rpm for different time periods of 5 to 240 min. The aliquots of the std‐BP dispersion were centrifuged at 5 krpm for 120 min. The resultant stock dispersions can be readily size‐selected to yield a small or large nanosheet, which is the primary advantage of this technique.^192^, ^193^, ^194^ The supernatant was subjected to controlled centrifugation with subsequently increasing rotation speeds of 2 krpm, 3 krpm, 4 krpm, 5 krpm, 10 krpm and 16 krpm, with each step performed for 2 h to yield samples with varying size distributions, including decreased sizes in the respective sediments. The main impact of this study is that the solvation shell of the solvent (CHP) molecules can protect the exfoliated nanosheets from reacting with water. **Figure** **11**a–e shows a photograph, low‐resolution TEM images and the low‐by‐pass bright‐field scanning transmission electron microscopy (STEM) image of the FL BP dispersion in CHP. A Butterworth‐filtered high‐angle annular dark field (HAADF) STEM image of the BP‐exfoliated dispersion in isopropanol, indicating the intact lattice, can be observed in Figure 11f.

Yasaei et al.^182^ examined various solvents from different chemical families such as alcohols, chloro‐organic solvents, ketones, cyclic or aliphatic pyrrolidones, N‐alkyl‐substituted amides and organosulfur compounds, which encompass a wide range of surface tensions (21.7 to 42.78 dyne cm^–1^) and polar interaction parameters (2.98 to 9.3 MPa^1/2^) to understand their performance in BP exfoliation. The bulk BP crystal (0.02 mg in 10 mL) was immersed in different solvents for a 15 h sonication (130 W, a total input energy of 1 MJ) and they found that aprotic and polar solvents such as DMF and DMSO as more suitable for atomically thin BP flakes, leaving stable and uniform dispersions.^195^ A 30 min centrifugation was performed at 2000 rpm, after which the supernatants with concentrations of up to 10 µg mL^–1^ were collected and dispersed in IPA. The dispersed flakes in the solution offer the best protection from degradation. Photographs of few‐layered BP dispersions in DMSO and DMF solvents after sonication, and after centrifugation and supernatant collection, are displayed in Figure 10a. Figure 10b shows AFM and SEM images of the BP nanoflakes on a SiO_2_/Si substrate where a “coffee‐ring” structure is observed in the low‐magnification SEM.

Woomer et al.^196^ surveyed the experimental conditions for liquid exfoliation and explored the first large‐scale production (10 g scale) of monolayer, bilayer and few‐layer phosphorus. The experimental process is described as follows: grounded BP was sonicated in anhydrous, deoxygenated organic liquids (isopropanol for 16 h) under inert atmosphere resulting in a change of color from black to reddish‐brown and finally to yellow that signifies a change in the electronic structure of BP. The color remains the same after few weeks with limited reaggregation, indicating the presence of small phosphorus particulates. They experimented with different solvents such as N‐methyl‐2‐pyrrolidone, 2‐propanol, cyclopentanone, 1‐cyclohexyl‐2‐pyrrolidone, 1‐dodecyl‐2‐pyrrolidinone, benzyl benzoate, 1‐octyl‐2‐pyrrolidone, 1‐vinyl‐2‐pyrrolidinone, benzyl ether, 1,3‐dimethyl‐2‐imidazolidinone, cyclohexanone, chlorobenzene, dimethylsulfoxide, benzonitrile, N‐methylformamide, dimethylformamide and benzaldehyde to understand their abilities in BP exfoliation. BP (10 mg) added to each solvent (20 mL) was sonicated (at 22 and 30 °C) for 13 h under anhydrous and air‐free conditions, which were centrifuged (at 3000 rpm for 30 min) further to remove unexfoliated BP. Benzonitrile was found to be a suitable candidate with a mean concentration of 0.11 ± 0.02 mg mL^–1^. Figure 11l–p shows a photograph, SEM image and TEM images; particularly noteworthy is the TEM image of a monolayer BP of an exfoliated BP suspension in isopropanol synthesized by Woomer et al.^196^

##### 4.2.2.2 Water‐Based Sonication

: Wang et al.^114^ used distilled water as the solvent, and it was bubbled with argon to eliminate the dissolved oxygen molecules to overcome the problem of oxidation in the sonication process. A scalable clean exfoliation with water of few‐layer BP was recently demonstrated by means of the tip sonication method.^173^ Bulk BP crystals were grounded to BP powders, the powders were dispersed in 20 mL of deionized (DI) water to obtain a concentration of 1 to 10 mg mL^–1^, and the dispersions were tip sonicated for 30 to 300 min. The supernatant was decanted from the settled dispersion after 12 h for centrifugation, yielding a BP nanosheet dispersion with a high concentration. The interesting finding of this study is that the BP nanosheets retain the high quality of the bulk crystals, with the excellent qualities of a very high crystallinity, an impurity‐free structure and stability in water. Lee et al.^197^ prepared few‐nm to <20 nm BP nanodots with an average diameter of ≈10 nm and a height of ≈8.7 nm, where BP (0.4 g, 12.8 mmol) was dispersed in distilled water (100 mL) for a 30 min sonication (20 kHz, 100 W). The supernatant liquid collected from the dispersion was ultrasonicated for 10 min and these steps were repeated two or more times to create smaller‐sized BP particles.

##### 4.2.2.3 Ionic‐Liquid‐Based Sonication

: Ionic liquids (ILs) are molten salts at RT that have been considered as green solvents for decades,^198^ with exciting properties such as non‐volatility, high thermal stability, high viscosity, high ionic conductivity, nontoxicity, versatile solubility and solvent recyclability compared to conventional organic solvents.^198^, ^199^ ILs are presented by researchers for efficient exfoliation of 2D materials to obtain a stable yield with a high concentration of the suspended nanoflakes.^200^, ^201^, ^202^, ^203^ ILs exhibit viscosities that are 1−3 orders of magnitude more than those of conventional organic solvents; this influences the rate of mass transport within the solution by preventing the BP nanosheets from restacking.^198^, ^200^ An environmentally friendly liquid exfoliation by means of ionic liquids was reported by Zhao et al.^200^ to exfoliate highly pure, crystalline, atomic‐scale and uniform mono‐ to few‐layer BP nanosheets by mild grinding and weak sonication. The first step in IL‐exfoliation was grinding bulk BP (30 mg) with ILs (0.5 mL) for 20 min using an agate mortar with a pestle that supplements the mechanical shear forces to reduce the exfoliation time due to the increase in the surface area of BP.^118^, ^182^, ^203^ The next step was to disperse the mixtures in ILs (3 mg mL^−1^ BP) by ice‐bath sonication (100 W) for 24 h to obtain suspensions of BP flakes which were further centrifuged at 4000 rpm for 45 min to remove the unexfoliated BP. The stable dispersions of BP nanosheets were obtained, with higher concentrations (≈0.95 mg mL^−1^) than the concentration (0.4 mg mL^−1^) obtained in NMP, and without sedimentation and aggregation.^183^

Zhao et al.^200^ employed nine ILs, which included 1‐butyl‐3‐methylimidazolium tetrafluoroborate ([BMIM] [BF_4_]), [BMIM] trifluoromethansulfonate ([TfO]), [BMIM] bis((trifluoromethyl)sulfonyl)imide ([Tf_2_N]), 1‐ethyl‐3‐methylimidazolium ([EMIM]) [Tf_2_N], [EMIM] [BF_4_], 1‐hexyl‐3‐methylimidazolium ([HMIM]) [BF_4_], 1‐octyl‐3‐methylimidazolium ([OMIM]) [BF_4_], 1‐hydroxyethyl‐3‐methylimidazolium ([HOEMIM]) [TfO] and [HOEMIM] [BF_4_]. Figure 11g shows a photograph of BP in [BMIM][TfO] and [HOEMIM][TfO] and the Tyndall effect of the diluted dispersions. It was found that all ILs employed were efficient in yielding highly concentrated BP nanosheet dispersions in which the coulombic force and π−π interactions between the aromatic ILs cations and the phosphorene layers are more significant in obtaining stabilized exfoliated BP nanoflakes.^201^ ILs with larger surface tensions yield higher‐concentration dispersions as a result of the easy break down in the interlayer van der Waals forces of bulk BP by the large surface tension, thereby preventing detached BP layers from restacking.^183^, ^200^, ^204^ The cations influence the concentration of the suspension by changing the cationic chain length, as observed from comparing [EMIM] [BF_4_], [BMIM] [BF_4_], [HMIM] [BF_4_], [OMIM] [BF_4_], and [HOEMIM] [BF4]. It was also demonstrated that the [HMIM][BF_4_], with a relatively low surface tension, gives rise to a high‐concentration suspension where the viscosity of [HMIM][BF_4_] (177 cP at 303 K) is higher than the viscosity of the other ILs (28−90 cP at 303 K). Electron microscopy studies of BP‐[HOEMIM][TfO] including the low‐magnification TEM image, the selected area electron diffraction (SAED) pattern, the HRTEM image and the magnified HRTEM image of selected areas are shown in Figure 11h–k. Hence, ILs are found to be more suitable solvents for the fabrication of atomically thin BP nanoflakes due to their strong cohesive dipolar nature and the planarity of the solvents.

##### 4.2.2.4 Tip‐Sonication‐Based Exfoliation

**Figure 12:**
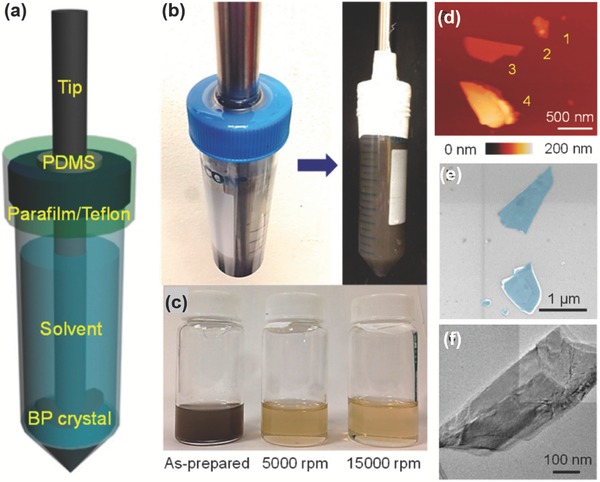
Tip ultrasonication exfoliation of BP in various solvents: Schematic (a) and photo with minimized exposure to ambient air (b) of the experimental setup. c) Photo of an ultrasonicated BP dispersion in NMP, 5000 rpm centrifugation and 15 000 rpm centrifugation (from left to right). d) AFM image of BP nanosheets on the SiO_2_/Si substrate, with different heights in N_2_ environment (1:16, 2:40, 3:29, and 4:128 nm). No evidence of bubbles or any other degradation is observed. e) False‐colored SEM image. f) Low‐resolution TEM image. Reproduced with permission.^183^ Copyright 2015, American Chemical Society.

: Kang et al.^183^ synthesized electronic‐grade BP dispersions using sealed‐tip ultrasonication at a reduced sonication time by means of anhydrous oxygen‐free organic solvents, thus avoiding the chemical degradation pathways for BP. The schematic and photo of the experimental setup are depicted in **Figure** **12**a,b. A sealed container lid was attached to an ultrasonicator tip/probe (0.125 in.) and driven at a higher power compared to conventional bath sonication to minimize the ultrasonication duration. Additionally, the interface between the tip and the lid was carefully sealed with PDMS, whereas Parafilm and Teflon tapes were used to seal the pathways between the lid and container to restrict O_2_ and H_2_O penetration. The synthesis was performed in an ice bath at ≈30 W power to obtain a BP concentration of ≈1 mg mL^–1^ in 1 h; on the other hand, bath sonication needs 15 to 24 h for the same exfoliation process.^182^, ^186^ To optimize the solvent, BP crystals were ultrasonicated under identical preparation conditions in acetone, chloroform, hexane, ethanol, IPA, DMF, and NMP, and the samples were opened only in an Ar glovebox to minimize O_2_ and H_2_O contamination. The obtained dispersions were further centrifuged at different speeds (500 to 15000 rpm) for 10 min to tune the size distribution of the solvent‐exfoliated BP nanosheets, resulting in the solution color changing from brown to yellow depending on the centrifugation speed (see Figure 12c). They confirmed a monotonic increase in the BP concentration with an increase in boiling point and surface tension; which agrees well with graphene.^188^ According to their results, NMP was found to be the optimal solvent to achieve stable BP dispersions. The light yellow solution has the most dilute concentration (≈0.01 mg mL^–1^) of BP nanosheets, which confirms the correlation between the centrifugation speed and the BP concentration. Moreover, the flake thickness and lateral size were also observed to decrease with increasing centrifugation speeds, and the BP dispersions centrifuged at 500 rpm yield thick BP nanosheets (>50 nm thick). Conversely, centrifugation speeds of 10000 and 15 000 rpm minimize the lateral size of the BP nanosheet in comparison with the BP dispersions centrifuged at 5000 rpm, giving rise to a relatively lower lateral area for the higher centrifugation speeds. Figure 12d–f presents AFM, SEM and TEM images of the exfoliated BP nanosheets on a SiO_2_/Si substrate. Although probe sonication and bath sonication are commonly used in the exfoliation of 2D layered materials, bath sonication was reported to be more efficient than probe sonication and the use of either one of them may result in the formation of irregular BP nanosheets.^118^, ^186^ Therefore, highly dispersed suspensions of ultrasmall BP QDs with a lateral size of 2.6 nm and a thickness of ≈1.5 nm were fabricated by the LPE technique using probe sonication and bath sonication in NMP.^205^ In this report, commercially available bulk BP crystals were used directly as the starting material without any prior grinding. BP (25 mg) added to NMP (25 mL) was first tip sonicated (1200 W power) for 3 h. The collected dispersion was ice‐bath sonicated (300 W) continuously for 10 h. The dispersion was centrifuged at 7000 rpm for 20 min and the collected supernatant solution with BP QDs was further centrifuged at 12 000 rpm for 20 min. A schematic of the detailed synthesis steps and AFM, TEM and HRTEM images of the obtained BP QDs are shown in Figure 9a–f. The BP QDs were dispersed in water, after centrifugation, and polyethylene glycol (PEG) was conjugated to them to enhance their stability.^205^

#### 4.2.3 Ultrasonication Mediated by Mechanical Milling

Sofer et al.^110^ ground bulk BP crystals in an agate mortar (10 min), ultrasonicated in DMF, NMP bis(2‐methoxyethyl) ether (DIGLYM) and acetonitrile (AN), and then milled at different times (up to 6 h) and milling speeds. The resultant BP nanoparticles (NPs) were separated by centrifugation and the average size of the colloidal particles (80–200 nm) depended on the milling speed, time and solvent. The solvent used significantly influenced the yield of the milling procedure, reaching 33 wt% for DIGLYM, 36 wt% for DMF, 47 wt% for NMP and 66 wt% for AN. Although AN gave the highest yield of BP nanoparticles, the long term stability of the BP nanoparticles produced was limited. This could be improved by transferring the BP nanoparticles from AN to DMF to form a stable colloidal solution.^110^ The removal of larger particles and the improvement of the particle size distribution was attained by centrifugation (at 5000 rpm and 10 000 rpm). The smallest BP quantum dots were obtained in AN and DMF at a centrifugation speed of 10 000 rpm. Sofer et al.^110^ demonstrated a particle size distribution of approximately 15 nm (minima) to 20–30 nm (maxima).

#### 4.2.4 Shear‐Assisted Exfoliation

**Figure 13:**
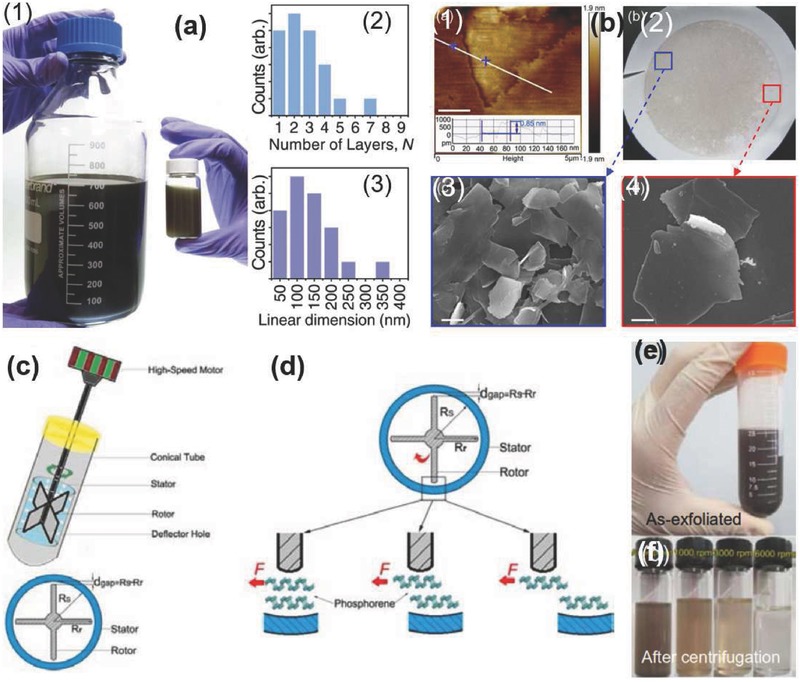
a) Scaled‐up synthesis of shear mixing and sonication of 2D phosphorus: 1) Photographs of exfoliated solutions from 6 g of BP and 800 mL of NMP (left image) to yield a highly concentrated suspension with thin pieces (right image) by centrifuging 40 mL of the above‐mentioned mixture at 20, 200 g. The size distribution is shown in (2) and (3). Reproduced with permission.^196^ Copyright 2015, American Chemical Society. b–f) Shear‐exfoliated phosphorene nanoflakes: b) AFM and SEM characterization. 1) AFM height measurement of a monolayer BP on the SiO_2_/Si substrate. Inset: height profile correlates to the white line. The stage height (≈0.85 nm) is evidence of monolayer phosphorene. Scale bar: 200 nm. 2) Photograph. 3, 4) SEM images of as‐filtrated phosphorene film on PTFE membrane with different size and dimensionality. Scale bar: 2 µm. c) Schematic of experimental setup for exfoliating bulk BP. d) Schematic of shear‐exfoliation in the laminar flow regime. e) Photograph of as‐exfoliated phosphorene in NMP before centrifugation. f) Photograph of phosphorene in NMP after 1000 rpm, 3000 rpm and 6000 rpm centrifugation. Reproduced with permission.^207^ Copyright 2016, IOP.

Shear‐assisted exfoliation is one of the LPE techniques adopted in the exfoliation of 2D materials.^135^, ^192^, ^196^, ^206^, ^207^, ^208^ Woomer et al.^196^ exfoliated large‐scale BP (10 g) by a shear mixing process using a shear mixer with square holes at RT, and in oxygen‐free and water‐free conditions, by bubbling nitrogen gas into the mixing container. They used two different types of BP (crystalline and polycrystalline) and NMP (specific grade)^192^ as the solvent. The high‐quality crystalline BP with millimeter‐sized crystals was difficult to grind but the low‐quality polycrystalline BP with trace amounts of red phosphorus was easy to grind. The low‐quality material could be successfully exfoliated by shear mixing irrespective of the conditions of shear mixing and the grade of NMP. Specifically, the separation of the layers is nucleated at the grain boundaries or at other defects in the material. High‐quality pulverized BP (6 g) was mixed with NMP (100 mL) and bath sonicated for 2 h. NMP (700 mL) was added to the sample and shear mixing was performed at 5000 rpm for 4 h. The dispersion was sonicated again for 3 h, shear mixed at 5000 rpm for 1 h and centrifuged. It was reported that 25% of the samples obtained by the large‐scale shear mixing process were monolayers with a lateral size comparable to that produced by small‐scale bath sonication.^196^ **Figure** **13**a shows details of the scaled production method, with photographs of the exfoliated solutions from 6 g of BP and 800 mL of NMP used to yield a highly concentrated suspension with thin pieces by centrifuging 40 mL of the above‐mentioned mixture at 20200 g. The size distribution is also shown.

Xu et al.^207^ demonstrated a shear exfoliation technique by making use of shear force to break down the interlayer van der Waals forces in appropriate solvents for exfoliation of monolayer or few‐layer phosphorene nanoflakes. A schematic of the experimental setup for exfoliating bulk BP, a schematic of shear‐exfoliation in the laminar flow regime, and AFM and SEM images of the exfoliated flakes are shown in Figure 13. The shear‐exfoliation process was carried out in an argon atmosphere with the BP immersed in NMP placed in a conical tube inside a glove box. The glove box contained a mixing head with a narrow gap (*d* = 0.2 mm) between the rotor and stator in which the high speed of rotor (*N*) caused a high shear rate (γ) within the gap. The turbid dispersion, obtained from shear exfoliation with a predetermined time and rotation speed, was purified by centrifugation for the removal of larger unexfoliated BP crystal lumps. The different centrifugation speeds produced phosphorene dispersions that were brown to pale yellow in colour. Consequently, the dispersions were filtered using a polytetrafluoroethylene (PTFE) membrane and were rinsed thoroughly with IPA for further removal of the NMP residual. The shear rate is a crucial parameter in shear exfoliation. The equation of shear rate can be given as:^207^

(1) $$\gamma \;\approx \pi N\left(2R_{r}\right)/d_{\mathrm{gap}}$$

where *N*, *R*_r_, *d*_gap_ are the rotor speed, rotor radius, and rotor‐stator gap, respectively. The shear rate increases proportionally with the rotor speed if the mixing head of the rotor‐stator is fixed (*R*_r_ = 16 nm, *d*_gap_ = 0.2 mm). The rotor speed (above a minimum of ≈1500 rpm) yields high‐quality phosphorene nanoflakes with a minimum shear rate of ≈1.25 × 10^4^ s^–1^. The Reynolds number (Re) can be expressed as:

(2) Re≈N2Rr2(ρ/η)

where ρ and η are the volume and viscosity of the NMP solvent, respectively; the minimum shear rate can be obtained within the laminar flow regime (Re < 10^4^).^207^ Phosphorene nanoflakes were successfully produced by a Jiuyang kitchen blender with rotating blades (rotation speed of ≈15 500 – 22 000 rpm), resulting in a fully developed turbulence (Re > 10^4^).^207^ Figure 13e,f depicts a photograph of the as‐exfoliated phosphorene in NMP before and after centrifugation (1000 rpm, 3000 rpm and 6000 rpm centrifugation).

### 4.3 Other Methods

#### 4.3.1 Plasma‐Assisted Fabrication

**Figure 14:**
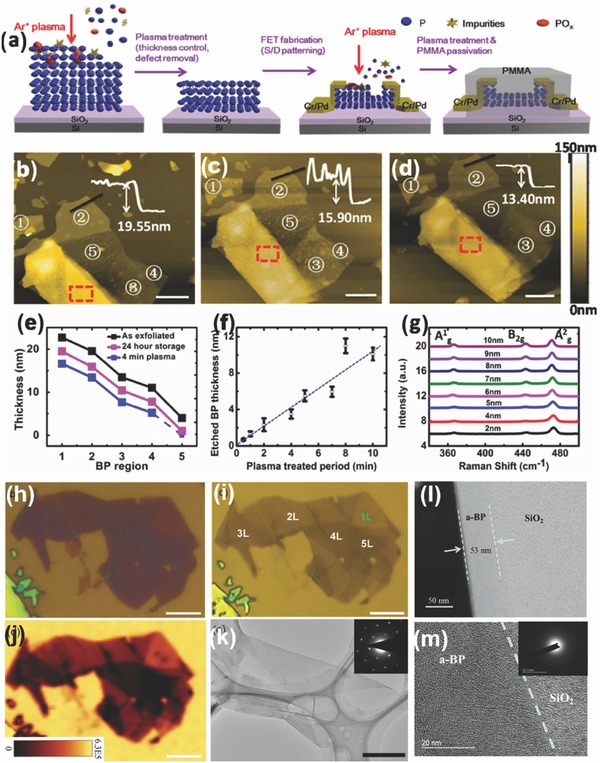
a–g) Plasma etching assisted fabrication: a) Illustration of the effects of the plasma treatment on the BP flake: thickness control, surface defect removal and device fabrication process. b–d) AFM mapping images of b) as‐exfoliated BP, c) BP after 24 h of storage and d) BP after plasma treatment for 4 min. Scale bars: 2 µm. e) Thickness of BP in different regions (1 to 5) in (b), (c) and (d). f) Etched BP thickness as a function of the plasma treatment time. g) Raman spectra of BP flakes of varying thickness. Reproduced with permission.^212^ Copyright 2015, American Chemical Society. h–k) Plasma thinning fabrication: Optical images of h) multilayered pristine phosphorene and i) the same flake after Ar^+^ plasma thinning. j) Reflection image of plasma‐treated flake in h). Scale bars: 5 µm. k) TEM image. Inset: SAED pattern. Scale bar: 500 nm. Reproduced with Permission.^211^ Copyright 2014, Springer. l,m) Fabrication by pulsed laser deposition: l) Cross‐sectional TEM images. m) High‐resolution TEM image. Inset: SAED showing amorphous phases. Reproduced with permission.^213^

Layer‐by‐layer thinning of multilayers of 2D materials under plasma or laser irradiation is an emerging approach for obtaining 2D monolayers.^209^, ^210^ Lu et al.^211^ demonstrated mechanical cleavage and plasma thinning for the effective fabrication of monolayer phosphorene. The few‐layer phosphorene realized with mechanical cleavage was transferred to a Si substrate coated with a SiO_2_ capping layer (300 nm); the film was further thinned down to phosphorene using an Ar^+^ plasma source (power of 30 W) at a pressure of 30 Pa for 20 s at RT. **Figure** **14**g–k shows an optical image of the multilayered pristine phosphorene flake before and after Ar+ plasma thinning and the TEM image with the SAED pattern. Figure 14g depicts the Raman peaks observed in bulk and exfoliated BP because of the vibrations taking place in the crystalline lattice of BP. After the process of plasma thinning, there was a decrease in the sample's optical contrast in which a single layer was also formed in a certain region. This plasma‐thinning technique proved to be strongly controllable for the realization of monolayer or few‐layer BP that can also be explicitly seen from TEM image morphology indicating highly crystalline BP.

Jia et al.^212^ adopted a plasma‐etching treatment not only to control the thickness of BP flakes but also to remove the chemical degradation caused on exposed oxidized BP surfaces. The thickness control of few‐layer BP was tuned by the plasma etching time. BP flakes (4‐ to 50‐nm thick) obtained from mechanical exfoliation was subjected to an Ar^+^ plasma treatment with different values for the plasma power (325 W, 350 W, 375 W, 400 W) at a pressure of 30 mTorr for 5 min to find the optimal plasma treatment condition (350 W), without causing damages to their morphology and crystal structure. Figure 14a explains the illustration of the effects of plasma treatment on a BP flake: the thickness control, surface defect removal and device fabrication process. The top surface had uniformly distributed protuberances or defects after exposure to atmosphere, and the defects intensified by merging into wider and taller bubbles with decreased density after 24 h storage at 10^–1^ Torr; this factor could be a major problem for the performance of devices. AFM mapping images of as‐exfoliated BP, after 24 h of storage and after plasma treatment for 4 min, are shown in Figure 14b–d. The plasma treatment (Ar^+^, 350 W, 4 min) on the same sample completely removed the bubbles found on the top surface.^212^ There was a uniform decrease (≈6 nm) in the BP thickness after the plasma etching process (for 4 min). It was also confirmed that the BP thickness can be significantly controlled by varying the plasma treatment duration. The BP thickness of different regions, the etched BP thickness as a function of the plasma treatment time and the Raman spectra of BP flakes of varying thickness are presented in Figure 14e–g.

#### 4.3.2 Pulsed Laser Deposition

Amorphous BP (a‐BP), a highly disordered form with a strong resemblance to BP and a thickness of 2 to 10 nm, was realized using conventional pulsed laser deposition (PLD) at a temperature as low as 150 °C, in contrast to the high‐temperature and high‐pressure growth techniques.^213^ The distance between the target of the bulk BP crystal and the substrates was maintained at 4 cm. The growth chamber was evacuated to a pressure of ≈1.5 × 10^−7^ Torr before proceeding to the PLD process. Cross‐sectional TEM images and a high‐resolution TEM image of the PLD deposited films, with an SAED pattern showing the amorphous phases, are shown in Figure 14l,m. The BP target was ablated by a KrF pulsed laser (λ = 248 nm) with a 5‐Hz repetition rate and the substrate temperature was kept at 150 °C. To obtain uniform a‐BP film growth, the BP target and the substrates were rotated during the deposition. Finally, the system containing the as‐deposited a‐BP ultrafilms was cooled down naturally to RT in the presence of high vacuum. **Table** **4** presents the studies of BP obtained by plasma‐based techniques.

**Table 4:** Other Methods

Method	Parameters	Substrate	Substrate thickness (nm)	BP Thickness (nm)	Device structure	Reference
Plasma Thinning	Ar^+^ plasma source (30 W power), pressure (30 Pa), 20 s at RT	SiO_2_/Si	300 (SiO_2_)	up to 5L	–	211
Plasma Etching	30 mTorr for 5 min, 350 W	SiO_2_/Si	285 (SiO_2_)	4 to 50 (as‐exfoliated), 2 to 10 (after plasma etching)	FET	212
PLD	KrF pulsed laser (λ = 248 nm), 5 Hz repetition rate, 150 °C 1.5 × 10^−7^ Torr	SiO_2_/Si	–	2 to 10 nm (a‐BP)	FET	213

## 5 Ways to Protect Phosphorene and Effective Passivation

The study of BP towards achieving device fabrication is challenging as a result of its fast degradation when exposed to ambient conditions. Hence, this problem has been addressed in literature by different researchers and will be discussed in this section. The electrostatics and structural buckling could affect the stable bonding configurations, which further influence the reduced chemical stability of 2D materials.^214^ Therefore, the ambient stability is a major factor to be considered in BP, as the phosphorus atoms have free lone electron pairs and valence bond angles of 102°.^215^ Bridgman^103^ observed oxidation of BP to phosphoric acid under humid atmospheric conditions. The irreversible BP oxidation into phosphate derivatives can be better understood from structural modifications such as the bubble formation in the BP flakes that completely ruins the high‐performance electronic properties of BP.^183^ The formation of pits and bubbles in bulk BP was also reported during scanning tunneling microscopy studies.^216^ Published reports have also demonstrated that few‐layered BP obtained by mechanical exfoliation degrades in air.^22^, ^150^, ^156^ Interestingly, the stability tests related to the response of BP film sensors were shown to be nearly unchanged after prolonged exposure (up to 3 months) in ambient conditions.^195^ The issue of chemical degradation of the thin BP flakes in ambient conditions is a major concern because it will cause a major impact in the electronic properties, thereby influencing device performance. This is the current major research problem.^22^, ^86^, ^155^, ^156^ As a result of effective passivation techniques of encapsulation adopted in semiconductor industry, devices based on carbon nanotube,^217^ and graphene^218^, ^219^ showed improved performance; therefore, passivation studies on BP based devices have increased.^36^, ^54^, ^220^ Atomic layer deposition (ALD)‐derived dielectrics were found to be compatible with BP FETs for the purpose of passivation.^21^, ^36^, ^220^

### 5.1 Theoretical Studies on Stability of Phosphorene

**Figure 15:**
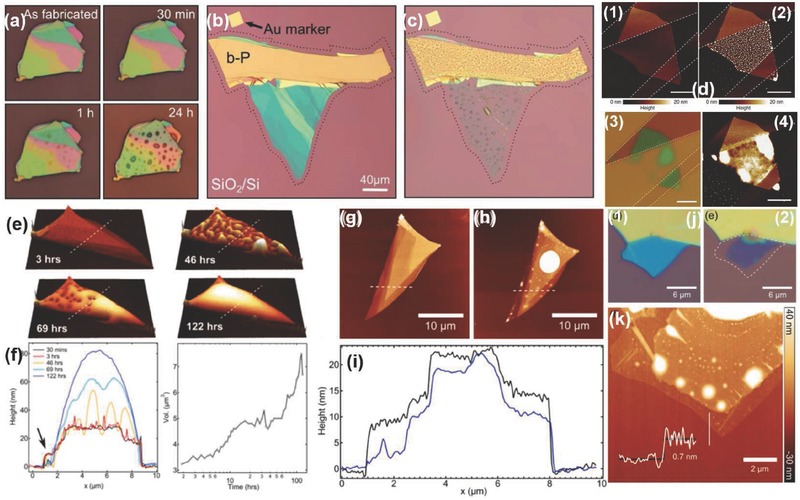
a–c) Aging of BP flakes: a) Optical images taken at different times after BP flake transfer; there is evidence of droplet‐like structures on the surface after one hour which grow when exposed to air. b,c) Comparison of sample immediately after the transfer and after two weeks in air, respectively. Reproduced with permission.^156^ Copyright 2014, IOP. d) AFM study of passivated and exposed ultrathin BP in ambient conditions: 1,2) AFM images of 5‐nm‐thick BP partly covered with graphene (shown by white dashed lines) taken after 10 min and 24 h exposure, respectively. 3) Optical image and 4) AFM image of BP/graphene after 48 h in ambient conditions. Scale bars: 4 mm. Reproduced with permission.^230^ Copyright 2015, Nature Publishing Group. e–k) Studies on environmental instability of exfoliated BP: e) AFM images acquired from BP flake kept in air at 3 h, 46 h, 69 h and 122 h after exfoliation. f, left) Line profiles taken across the BP flake indicated by the dotted lines from the scans of (e) and for scan taken immediately after exfoliation. f, right) Total volume of flake and water during the measurement period. g) AFM image taken ≈30 min after exfoliation for the same flake in (e). h) AFM image of the same flake after exposure and pumping/heating. White saturated region and white dotted lines represent left‐over residue and location of the line profile shown in (i). i) AFM height scans performed at the dotted lines in (g) and (h). Black and blue curves show the height before and after exposure. j1) Optical image of another flake immediately after exfoliation and before 5 days of ambient exposure. j2) Same flake in (1) after exposure and pumping/heating. Edge of the original flake is marked as a white dotted line. k) AFM image of (j2). Location of the line profile in the inset is shown as a white line (white curve). Reproduced with permission.^155^ Copyright 2015, IOP.

The thickness‐dependent photoassisted oxidation reaction of phosphorene with oxygen dissolved in adsorbed water was found to be related to intrinsic defects, and coating the flake with a capping layer of 300 nm of parylene C provided a barrier against water diffusion and prevented degradation.^150^, ^221^ Favron et al.^150^ proposed a model in which the rate of oxidation depends linearly on the oxygen concentration and the light intensity, and exponentially on the square of the energy gap. The detailed process of three‐step exfoliation with PDMS is shown in Figure 5e: (1) exfoliation on PDMS‐1, (2) flakes were rolled on a semi‐spherical PDMS‐2 stamp and (3) the stamp was rolled on a SiO_2_/Si substrate with an estimated speed of 0.1 cm s^–1^. Wang et al.^222^ presented an atomic level understanding of the stability of phosphorene by DFT calculations with first‐principles molecular dynamics (MD) simulations with respect to the interaction of phosphorene with O_2_ and H_2_O. The theoretical and experimental studies proved that O_2_ can easily dissociate on phosphorene to form the oxidized lattice at room temperature.^183^, ^223^, ^224^ The exothermic energy (Δ*Q*) of O_2_ dissociation on black phosphorene is 4.46 eV per O_2_ molecule.^222^ It was also found that H_2_O will not interact directly with the pristine phosphorene lattice because it prefers to bind to the surface of phosphorene through hydrogen bonds. In addition, H_2_O will interact exothermically with phosphorene if it has first been oxidized. Hence, it was concluded that the most likely route for the chemical degradation of the phosphorene‐based devices in air is first through oxidation and then, by an exothermic reaction with water. The stability of phosphorene in air is also highly dependent on humidity. Castellanos‐Gomez et al.^156^ studied the environmental stability of BP flakes and showed that they are very hydrophilic, and long term exposure (of more than a week) to the moisture in air etches the thin parts of the flakes. They have attributed the presence of droplets on the surface of the flakes to adsorbed water (see **Figure** **15**a–c). There are also reports confirming the stability of phosphorene in the presence of H_2_O.^225^, ^226^ DFT calculations of Wang et al.^222^ confirmed that the H_2_O molecule will not interact strongly with the pristine phosphorene.

### 5.2 Encapsulation with Solvent

The LPE of BP becomes problematic due to the degradation caused by the instability of BP in oxygen or water and stabilization is required.^35^, ^36^, ^156^ The encapsulation of BP was proposed as a possible solution to protect its reaction with environmental species.^36^, ^220^ The solvent choice is important in reducing the BP oxidation in LPE because the solvation shell functions as a barrier to prevent oxidative species from approaching the surface of the nanosheet/nanoflake.^191^ Computational studies by Hanlon et al.^191^ showed that the degradation occurs by the reaction of water molecules with BP, causing the removal of phosphorus atoms and leading to the formation of phosphine and phosphorous acid; tis degradation was only observed at the nanosheet edge, as noticed in TiS_2_.^227^ Furthermore, the degradation of the BP nanoflakes during the reaction with water occurs predominantly at the nanosheet edges exposed to ambient conditions. Hence, examining the oxidation of black phosphorus nanosheets of varying sizes is helpful for understanding the degradation mechanism. A reduction in the absorbance value over time was found in all the samples, confirming the degradation of few‐layer BP.^191^ The stability was also influenced by the size of the BP flakes, where the large flakes are more stable than the smaller ones.

Favron et al.^54^ presented a systematic investigation of the chemical reactivity of BP using in‐situ Raman and transmission electron spectroscopy, and highlighted the process responsible for the origin of the irreversible thickness‐dependent photo‐induced oxidation reaction by the major environmental parameters of water, oxygen and visible light. Their studies proposed guidelines for preventing oxidation and explained the methods for obtaining pristine (i.e., oxygen‐free) monolayers. AFM studies performed shortly after exfoliation revealed a homogeneous distribution of small bumps or defects at the surface of the layer that seemed to collapse from the top of layer (rather than from the edges) indicating the degradation mechanism in similar with that observed in previous reports.^22^, ^156^ After a few days, a drastic transformation along with the formation of new droplets was noticed near the initial position of the flakes. They discovered an interesting phenomenon of significant reduction in the degradation when the samples were placed in the dark. To understand this, they performed experiments in vacuum (<5 × 10^–6^ Torr) and the results show no evidence of degradation. The light‐induced oxidative reaction mechanism includes two main reaction steps with the coverage (θ) of a pristine and intrinsic 2D phosphane given as:^54^

(3) θ+hν ⇌ θ∗

(4) $$\theta *+O_{2\left(\mathrm{aq}\right)}\to \;O_{2\left(\mathrm{aq}\right)}^{-}+\theta \to \theta^{\mathrm{ox}}$$

The optical excitation of the 2D‐phosphane ground state gives rise to a coverage of excited 2D‐phosphane (θ*), in which the steady‐state population of phosphane depends on the photon flux, the recombination rate and the absorption cross section (Equation (3)). According to Equation (4), the excited state of the population θ* experiences a charge transfer reaction with the aqueous oxygen molecules adsorbed at the surface of the layer, causing p‐doped 2D phosphane. The reactive intermediate species (i.e*.*, the strong Brønsted bases), such as the superoxide anions, are provoked by the charge transfer reaction with the oxygen–water redox couple and subsequently react with the 2D‐phosphane layer's surface atoms to etch the surface, resulting in the coverage oxidized species θ^ox^. Favron et al.^54^ concluded that the layer thickness, oxygen concentration and light intensity played a major role in controlling the reaction rate, in which the oxidation rate varied exponentially with the square of the layer's energy gap. Woomer et al.^196^ proposed a rapid liquid exfoliation approach for obtaining crystalline and unoxidized monolayer and few‐layer BP flakes (pristine), and these flakes were subjected to rapid oxidation when exposed to air. All the exfoliation and centrifugation experiments were carried out in an inert atmosphere.

### 5.3 Encapsulation with ALD‐Derived Al_2_O_3_

Exfoliated BP flakes, without encapsulation, can be easily chemically degraded upon exposure to ambient conditions. The studies of Wood et al.^36^ confirmed that the irreversible reaction of BP with O_2_‐saturated H_2_O was responsible for the oxidized phosphorus species (PO_x_), thereby providing an explanation for the structure and chemistry of degradation process.^54^, ^216^, ^228^ They found small topographic protrusions (bubbles) above the plane of the BP flakes shortly after exfoliation, whereas the bubble density and the height of the bubbles increased after 1 day of ambient exposure. The density of the bubbles decreased to form wider and taller bubbles upon an increased ambient exposure irrespective of the thickness of the flake. The wetting character of the underlying substrate can be modified to change the surface diffusion rate of oxygenated H_2_O and control the degradation of BP.^36^ The hydrophobic substrates allow more degradation compared to hydrophilic substrates because oxygenated H_2_O diffuses more rapidly on hydrophobic surfaces.^229^ ALD‐derived aluminum oxide (AlO_x_)‐encapsulated BP did not have degradation‐related bubbles after ambient exposure and there was no increase in the root mean square roughness of the surface with time. This would preserve the high carrier mobilities and the *I*_ON_/*I*_OFF_ ratios in BP FETs.^36^, ^183^ Al_2_O_3_ grown on the top of transistors causes the polarity to change from p‐type to ambipolar, which is attributed to the change in the Schottky barrier heights of the electrons and holes present at the metal contact edges from the fixed charges in the ALD dielectric.^220^

### 5.4 Passivation with Graphene and Boron Nitride

The first‐principles calculations of the surface reactions with oxygen, which cause the degradation of phosphorene, showed a mechanism for phosphorene oxidation in which dangling oxygen atoms increase the hydrophilicity of phosphorene and allow the easy introduction of oxygen defects under normal environmental conditions.^223^ By avoiding the contact of oxidized samples with water, the formation of complex defects that lead to sample deterioration can be prevented. Interestingly, graphene and hexagonal boron nitride (hBN) are excellent gas barriers, with their ultrathin nature resulting in minimum interference with the properties of the underlying crystal.^230^, ^231^, ^232^, ^233^ Therefore, Doganov et al.^230^ presented a means for the passivation of BP by using atomically thin graphene and hBN in an inert Ar gas environment to protect the pristine BP crystals upon exposure to ambient air and fabrication chemicals. The unprotected BP surface exhibited significant roughness compared to that of BP passivated with graphene or hBN, and the passivated regions showed degradation near the edges after a 48 h exposure in ambient conditions. The oxygen trap states or the chemical byproducts of the oxidation on the top surface may cause the p‐doping and low electron conduction observed in non‐passivated BP devices.^230^ The long term stability achieved by multi‐layer graphene (1L, 2L, 3L) passivation could be a great choice for large‐area coverage to achieve an enhanced protection of BP in practical applications.^234^ Kim et al.^234^ suggested that the direct growth of graphene on BP would provide a tight bond and overcome the nano‐gap that exists between BP and graphene in the conventional transfer‐passivation technique, thereby preventing the attack of air.

### 5.5 Passivation with Ti Metal Substitution and SiO_2_

Passivation with Ti metal substitution can provide an effective solution for the application of layered BP by improving the stability of the material. The substitution of Ti atoms into BP atomic lattices provided a resistance of the system to humidity and oxygen due to the robust stability of the BP@TiO_2_ hybrid system.^22^, ^235^ The formation mechanism of BP@TiO_2_ is given in equations (5) and (6) where Ti(O_4_C_4_H_9_)_4_ hydrolyzes rapidly in presence of water to give Ti(OH)_4_ on the surface of few‐layer BP. Finally, Ti‐OH aggregates to form Ti—O—Ti or Ti—O(H) bonds.

(5) $$\mathrm{Ti}-\mathrm{OH}+\mathrm{HO}-\mathrm{Ti}\to \mathrm{Ti}-O-\mathrm{Ti}+H_{2}O$$

(6) $$\mathrm{BP}\;\mathrm{surfaces}\;-P\;-\mathrm{OH}+\mathrm{HO}\;-\mathrm{Ti}\;\to \mathrm{BP}\;\mathrm{surfaces}\;-P\;-O-\mathrm{Ti}+\;H_{2}O$$

The passivation of the mechanically exfoliated BP flakes with a low‐temperature deposited SiO_2_ layer (100 nm) resulted in enhanced environmentally stable BP FETs.^236^ The SiO_2_‐passivated devices showed high on/off current ratios (of over 600) even after one week of exposure; this is lower than the initial value (810).

### 5.6 Study of Potential Structural Modification in BP

Kang et al.^183^ demonstrated a scalable method for synthesizing pristine 2D BP nanosheets by LPE in organic solvents, in which the sealed‐tip ultrasonication system enables the exfoliation of BP into anhydrous, oxygen‐free solvents, thereby preventing the known chemical degradation pathways. In situ sputtering was also employed to eliminate oxygen defects present in both the solvent‐exfoliated BP nanosheets and bulk BP crystals. Moreover, the residual NMP from the LPE also quenches the oxidation rate of BP in ambient conditions. Kang et al.^183^ fabricated five different samples on 300 nm SiO_2_/Si substrates by (i) mechanical exfoliation, (ii) liquid exfoliation in NMP, (iii) mechanical exfoliation with 1 h dipping in NMP, (iv) ALD alumina‐encapsulation by solvent exfoliation in NMP and (v) ALD alumina‐encapsulation by mechanical exfoliation to study the potential structural modification of BP in ambient conditions. All the samples were stored in dark, ambient conditions with an average temperature of 26.9 °C. There was no evidence of degradation (bubbles) in all the samples shortly after their preparation. Bubbles were apparent on the surface of the mechanically exfoliated BP after 1 day of exposure in ambient conditions in agreement with previous reports.^36^ Bubbles were also observed on the solvent‐exfoliated BP sample and the mechanically exfoliated BP sample with 1 h NMP after 2 days of exposure, irrespective of their flake thickness or lateral size. The bubbles on the first three samples grew larger and taller after 7 days. It was found that the residual NMP prevents BP degradation for approximately 24 h of total ambient exposure because of the NMP encapsulation or intercalation. The solvent‐exfoliated and mechanically exfoliated BP samples passivated with the ALD alumina overlayers do not show any degradation bubbles even after 7 days. The comparative studies of the solvent‐exfoliated BP nanosheets with mechanically exfoliated BP as a function of the ambient exposure time showed that BP nanosheets with a similar oxide content demonstrated slow ambient degradation kinetics for solvent‐exfoliated BP nanosheets than for mechanically exfoliated BP after 1, 2, 3 and 7 days of ambient exposure.^36^, ^183^

### 5.7 Stability of BP in Different Environments

The important issue of BP degradation has kindled research in this area. The thinner flakes were found to absorb water faster than the thicker ones, with a layer‐by‐layer etching observed in ambient conditions.^155^ Figure 15e–k shows AFM images acquired from a BP flake kept in air at 3 h, 46 h, 69 h and 122 h after exfoliation, The AFM image was taken ≈30 min from exfoliation before and after exposure and pumping/heating; the optical image of another flake was obtained to understand the environmental instability of exfoliated BP. Doganov et al.^230^ suggested that the top‐side encapsulation of hBN and graphene in BP FET devices can achieve passivation in BP. Theoretical studies showed that the surface of black phosphorus is intrinsically hydrophilic,^156^, ^237^ in contrary to another report where the surface becomes hydrophilic after oxidation.^223^ According to Favron et al.,^150^ water, oxygen and visible light are the three major environmental parameters that are simultaneously responsible for the BP degradation. The humidity sensors fabricated by Yasaei et al.^195^ showed stability for more than 3 months, and their degradation studies showed faster degradation in humid air, which is in agreement with previous results.^36^, ^83^, ^155^, ^156^

**Figure 16:**
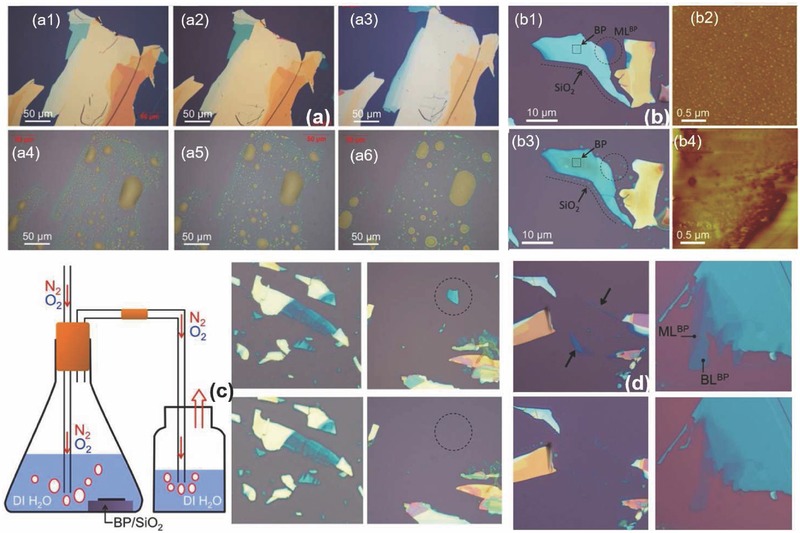
Role of oxygen and water in degradation of exfoliated‐BP: (a) Morphology change after exposure to water and air. a1) Optical image of freshly prepared BP flake. a2) Same flake after 1‐week immersion in DI water. a3) Same flake after 2‐weeks exposure to water. a4) After removing flake from DI water and exposing to air for 1 week, a complete dissolution of the flake occurs and droplet‐like residues are observed. a5) Same sample after annealing at 120 °C for 2 h in ultrahigh vacuum (10^–9^ Torr). a6) After additional annealing at 250 °C for 2 h in ultrahigh vacuum. b) Optical and AFM images. b1) Optical image of freshly exfoliated pristine BP flake. b2) AFM image of the area marked by the dashed box in (b1) within the flake. b3) Optical image after exposing sample to air for 1 day. Chain of islands/droplets are building up along the flake edge (e.g., dashed line) with the vanishing of entire monolayer BP segment in (b1) outlined by the dashed circle. b4) AFM image within the flake (dashed square). c) Effects of immersion in oxygen‐depleted and oxygen‐enriched DI water and d) effects of air exposure and water immersion. Reproduced with permission.^238^ Copyright 2016, American Chemical Society.

To obtain a clear picture of the chemistry involved in BP degradation, there is a need for experiments to propose suitable encapsulation strategies. Accordingly, Huang et al.^238^ carried out a systematic investigation of BP degradation in different environments of air, water with dissolved O_2_ and deaerated water. Their studies showed the rapid degradation of devices exposed to air and oxygen‐saturated water whereas the immersion in deaerated water showed stable morphologies. The stability in oxygen‐free water points to a stability in aqueous solutions (e.g., in photo‐catalysis or electrochemistry). There were no droplets observed even after the BP was taken out of DI water, suggesting that the surface of black phosphorus may be not intrinsically hydrophilic as was previously reported.^156^, ^237^ The observations of Huang et al.^238^ suggested that the droplet‐like objects were rather caused by an accumulation of species with a lower vapor pressure (phosphorus oxides) and these objects did not contain water. Although there are reports available showing an increase in the decomposition rate in thin flakes, no evidence was observed for such action in oxygen‐depleted conditions down to the monolayers.^150^, ^155^, ^238^ **Figure** **16** details the morphology change in exfoliated BP flakes after exposure to water and air, their optical and AFM images, the effect of immersion in oxygen‐depleted and oxygen‐enriched DI water and finally, the effect of air exposure and water immersion. Consequently, BP cannot activate and react with H_2_O itself but requires O_2_ present in air or dissolved in water as the primary pathway. The degradation of BP in ambient conditions is usually initiated by contact with oxygen, whereas water does not contribute significantly in the reaction.^238^

## 6 Summary and Outlook

Phosphorene has changed the landscape of many research areas in science and technology, particularly in condensed matter physics, and it has received much recent attention as the base component of novel nanodevices. Despite intense interest and an abundance of experimental success by device physicists, the widespread implementation of phosphorene is yet to occur. This is primarily due to the difficulty of reliably producing high‐quality samples, especially in a scalable fashion. Hence, the main focus of this review concerns the fabrication of layered BP, especially phosphorene, which may pave the way towards the realization of high‐performance nano‐devices. As phosphorene is the fundamental atomic layer of black phosphorus, the study of its fabrication techniques has become significant. The process involved in mechanical exfolaition method is difficult for obtaining uniform samples, as one can obtain flakes that have different layers which is also time‐consuming. The main challenge is that the performance of BP‐based devices depends not only on the number of layers but also on the quality of the crystal lattice. The quest for new exfoliating media is required to find environmental friendly solvents, thereby avoiding the use of toxic and impractical solvents. The study of BP towards device fabrication becomes more challenging because of its fast degradation when exposed to ambient conditions.

Beyond the progress in fabrication techniques of phosphorene, the advancement in facile synthesis routes is needed to facilitate theoretical studies. The large‐scale synthesis, accompanied by the large‐area growth and the control of the layer thickness of phosphorene, is the present need in device fabrication. Consequently, new bottom‐up methods can be developed to explore the benefits of phosphorene, which would be a great milestone for realizing advanced next‐generation devices in optoelectronics, electronics, sensors and photovoltaics based on this material. The salient features of monolayer‐ and few layer‐ black phosphorus can be well understood by fundamental studies on different factors such as the effect of strain, the nature and impact of defects and the effect of doping, quantum size effects and so on. The perspectives and interest in the study of the underlying physics in phosphorene would pave the way for the exploration of new interesting phenomena and make advancement in the area of two‐dimensional materials.

## References

[1] L. Wang , X. Ma , R. Chen , Y.‐Q. Yu , L.‐B. Luo , J. Mater. Sci.: Mater. Electron. 2015, 26, 4290.

[2] Y. Chen , G. Jiang , S. Chen , Z. Guo , X. Yu , C. Zhao , H. Zhang , Q. Bao , S. Wen , D. Tang , Opt. Express 2015, 23, 12823. 26074536 10.1364/OE.23.012823

[3] H. Chen , K. Liu , L. Hu , A. A. Al‐Ghamdi , X. Fang , Mater. Today 2015, 18, 493.

[4] Y. Jiang , W. J. Zhang , J. S. Jie , X. M. Meng , X. Fan , S. T. Lee , Adv. Funct. Mater. 2007, 17, 1795.

[5] S. Cho , M. S. Fuhrer , Phys. Rev. B 2008, 77, 081402.

[6] F. Xia , T. Mueller , Y.‐m. Lin , A. Valdes‐Garcia , P. Avouris , Nat. Nanotechnol. 2009, 4, 839. 19893532 10.1038/nnano.2009.292

[7] T. Mueller , F. Xia , P. Avouris , Nat. Photonics 2010, 4, 297.

[8] Y. Zhang , T. Liu , B. Meng , X. Li , G. Liang , X. Hu , Q. J. Wang , Nat. Commun. 2013, 4, 1811. 23651999 10.1038/ncomms2830

[9] K. Roy , M. Padmanabhan , S. Goswami , T. P. Sai , G. Ramalingam , S. Raghavan , A. Ghosh , Nat. Nanotechnol. 2013, 8, 826. 24141541 10.1038/nnano.2013.206

[10] W. J. Yu , Y. Liu , H. Zhou , A. Yin , Z. Li , Y. Huang , X. Duan , Nat. Nanotechnol. 2013, 8, 952. 24162001 10.1038/nnano.2013.219 PMC4249654

[11] A. N. Rudenko , M. I. Katsnelson , Phys. Rev. B 2014, 89, 201408.

[12] S. B. Lu , L. L. Miao , Z. N. Guo , X. Qi , C. J. Zhao , H. Zhang , S. C. Wen , D. Y. Tang , D. Y. Fan , Opt. Express 2015, 23, 11183. 25969214 10.1364/OE.23.011183

[13] J. Qiao , X. Kong , Z.‐X. Hu , F. Yang , W. Ji , Nat. Commun. 2014, 5, 4475. 25042376 10.1038/ncomms5475 PMC4109013

[14] Y. Takao , A. Morita , Physica B+C 1981, 105, 93.

[15] S. Morozov , K. Novoselov , M. Katsnelson , F. Schedin , D. Elias , J. Jaszczak , A. Geim , Phys. Rev. Lett. 2008, 100, 016602. 18232798 10.1103/PhysRevLett.100.016602

[16] Z. Ling , R. Yang , J. Chai , S. Wang , W. Leong , Y. Tong , D. Lei , Q. Zhou , X. Gong , D. Chi , Opt. Express 2015, 23, 13580. 26074606 10.1364/OE.23.013580

[17] P. Wu , Y. Dai , T. Sun , Y. Ye , H. Meng , X. Fang , B. Yu , L. Dai , ACS Appl. Mater. Interfaces 2011, 3, 1859. 21534599 10.1021/am200043c

[18] L. Li , X. Fang , T. Zhai , M. Liao , U. K. Gautam , X. Wu , Y. Koide , Y. Bando , D. Golberg , Adv. Mater. 2010, 22, 4151. 20730817 10.1002/adma.201001413

[19] P. Hu , J. Zhang , M. Yoon , X.‐F. Qiao , X. Zhang , W. Feng , P. Tan , W. Zheng , J. Liu , X. Wang , Nano Res. 2014, 7, 694.

[20] K. I. Bolotin , K. Sikes , Z. Jiang , M. Klima , G. Fudenberg , J. Hone , P. Kim , H. Stormer , Solid State Commun. 2008, 146, 351.

[21] H. Liu , A. T. Neal , Z. Zhu , Z. Luo , X. Xu , D. Tománek , P. D. Ye , ACS Nano 2014, 8, 4033. 24655084 10.1021/nn501226z

[22] S. P. Koenig , R. A. Doganov , H. Schmidt , A. C. Neto , B. Oezyilmaz , Appl. Phys. Lett. 2014, 104, 103106.

[23] M. Buscema , D. J. Groenendijk , G. A. Steele , H. S. van der Zant , A. Castellanos‐Gomez , Nat. Commun. 2014, 5, 4651. 25164986 10.1038/ncomms5651

[24] Y. Deng , Z. Luo , N. J. Conrad , H. Liu , Y. Gong , S. Najmaei , P. M. Ajayan , J. Lou , X. Xu , P. D. Ye , ACS Nano 2014, 8, 8292. 25019534 10.1021/nn5027388

[25] M. Engel , M. Steiner , P. Avouris , Nano Lett. 2014, 14, 6414. 25299161 10.1021/nl502928y

[26] T. Low , M. Engel , M. Steiner , P. Avouris , Phys. Rev. B 2014, 90, 081408.

[27] C. M. Park , H. J. Sohn , Adv. Mater. 2007, 19, 2465.

[28] V. Tayari , N. Hemsworth , I. Fakih , A. Favron , E. Gaufrès , G. Gervais , R. Martel , T. Szkopek , Nat. Commun. 2015, 6, 7702. 26151889 10.1038/ncomms8702 PMC4506510

[29] S. Cui , H. Pu , S. A. Wells , Z. Wen , S. Mao , J. Chang , M. C. Hersam , J. Chen , Nat. Commun. 2015, 6, 8632. 26486604 10.1038/ncomms9632 PMC4639804

[30] F. Schedin , A. Geim , S. Morozov , E. Hill , P. Blake , M. Katsnelson , K. Novoselov , Nat. Mater. 2007, 6, 652. 17660825 10.1038/nmat1967

[31] L. A. Falkovsky , Phys.‐Usp. 2008, 51, 887.

[32] K. Novoselov , A. K. Geim , S. Morozov , D. Jiang , M. Katsnelson , I. Grigorieva , S. Dubonos , A. Firsov , Nature 2005, 438, 197. 16281030 10.1038/nature04233

[33] W. Zhang , C.‐P. Chuu , J.‐K. Huang , C.‐H‐ Chen , M.‐L. Tsai , Y.‐H. Chang , C.‐T. Liang , Y.‐Z. Chen , Y.‐L. Chueh , J.‐H. He , Sci. Rep. 2014, 4, 3826. 24451916 10.1038/srep03826 PMC3899643

[34] N. Huo , S. Yang , Z. Wei , J. Li , J. Mater. Chem. C 2013, 1, 3999.

[35] L. Li , Y. Yu , G. J. Ye , Q. Ge , X. Ou , H. Wu , D. Feng , X. H. Chen , Y. Zhang , Nat. Nanotechnol. 2014, 9, 372. 24584274 10.1038/nnano.2014.35

[36] J. D. Wood , S. A. Wells , D. Jariwala , K.‐S. Chen , E. Cho , V. K. Sangwan , X. Liu , L. J. Lauhon , T. J. Marks , M. C. Hersam , Nano Lett. 2014, 14, 6964. 25380142 10.1021/nl5032293

[37] J. S. Kim , P. J. Jeon , J. Lee , K. Choi , H. S. Lee , Y. Cho , Y. T. Lee , D. K. Hwang , S. Im , Nano Lett. 2015, 15, 5778. 26274095 10.1021/acs.nanolett.5b01746

[38] E. Gibney , Nature 2015, 522, 274. 26085254 10.1038/522274a

[39] R. Hultgren , N. Gingrich , B. Warren , J. Chem. Phys. 1935, 3, 351.

[40] J. Wu , N. Mao , L. Xie , H. Xu , J. Zhang , Angew. Chem. 2015, 127, 2396. 10.1002/anie.201410108 25611334

[41] F. Xia , H. Wang , Y. Jia , Nat. Commun. 2014, 5, 4458. 25041752 10.1038/ncomms5458

[42] V. Tran , R. Soklaski , Y. Liang , L. Yang , Phys. Rev. B 2014, 89, 235319.

[43] T. Low , A. Rodin , A. Carvalho , Y. Jiang , H. Wang , F. Xia , A. C. Neto , Phys. Rev. B 2014, 90, 075434.

[44] S. Appalakondaiah , G. Vaitheeswaran , S. Lebegue , N. E. Christensen , A. Svane , Phys. Rev. B 2012, 86, 035105.

[45] G. Qin , Q.‐B. Yan , Z. Qin , S.‐Y. Yue , H.‐J. Cui , Q.‐R. Zheng , G. Su , Sci. Rep. 2014, 4, 6946. 25374306 10.1038/srep06946 PMC4221793

[46] J.‐W. Jiang , H. S. Park , J. Phys. D: Appl. Phys. 2014, 47, 385304.

[47] J. Zhang , H. Liu , L. Cheng , J. Wei , J. Liang , D. Fan , J. Shi , X. Tang , Q. Zhang , Sci. Rep. 2014, 4, 6452. 25245326 10.1038/srep06452 PMC4171703

[48] R. Fei , L. Yang , Nano Lett. 2014, 14, 2884. 24779386 10.1021/nl500935z

[49] J.‐W. Jiang , H. S. Park , Nat. Commun. 2014, 5, 4727. 25131569 10.1038/ncomms5727

[50] J.‐W. Jiang , 2015, arXiv:1503.00200.

[51] H.‐Y. Zhang , J.‐W. Jiang , J. Phy. D: Appl. Phys. 2015, 48, 455305.

[52] H. Lv , W. Lu , D. Shao , Y. Sun , Phys. Rev. B 2014, 90, 085433.

[53] R. Fei , L. Yang , Appl. Phys. Lett. 2014, 105, 083120.

[54] A. Favron , E. Gaufrès , F. Fossard , P. Lévesque , A. Phaneuf‐L'Heureux , N. Tang , A. Loiseau , R. Leonelli , S. Francoeur , R. Martel , 2014, arXiv:1408.0345 [cond‐mat.mes‐hall]. 10.1038/nmat4299 26006004

[55] Y. Mu , M. Si , Europhys. Lett. 2015, 112, 37003.

[56] L. A. Falkovsky , Phys.‐Usp. 2008, 51, 887.

[57] X. Wang , A. M. Jones , K. L. Seyler , V. Tran , Y. Jia , H. Zhao , H. Wang , L. Yang , X. Xu , F. Xia , Nat. Nanotechnol. 2015, 10, 517. 25915195 10.1038/nnano.2015.71

[58] R. W. Keyes , Phys. Rev. 1953, 92, 580.

[59] A. Brown , S. Rundqvist , Acta Crystallogr. 1965, 19, 684.

[60] Y. Maruyama , S. Suzuki , K. Kobayashi , S. Tanuma , Phys. B+C 1981, 105, 99.

[61] Y. Akahama , S. Endo , S.‐i. Narita , J. Phys. Soc. Jpn. 1983, 52, 2148.

[62] H. Asahina , A. Morita , J. Phys. C: Solid State Phys. 1984, 17, 1839.

[63] S. Zhang , J. Yang , R. Xu , F. Wang , W. Li , M. Ghufran , Y.‐W. Zhang , Z. Yu , G. Zhang , Q. Qin , ACS Nano 2014, 8, 9590. 25188827 10.1021/nn503893j

[64] X. Peng , Q. Wei , A. Copple , Phys. Rev.B 2014, 90, 085402.

[65] V. Wang , Y.‐C. Liu , Y. Kawazoe , W.‐T. Geng , J. Phys. Chem. Lett. 2015, 6, 4876. 26582362 10.1021/acs.jpclett.5b02047

[66] L. Li , J. Kim , C. Jin , G. Ye , D. Y. Qiu , F. H. da Jornada , Z. Shi , L. Chen , Z. Zhang , F. Yang , 2016, arXiv:1601.03103.

[67] Z. Guo , H. Zhang , S. Lu , Z. Wang , S. Tang , J. Shao , Z. Sun , H. Xie , H. Wang , X. F. Yu , Adv. Funct. Mater. 2015, 25, 6996.

[68] Q. Yao , C. Huang , Y. Yuan , Y. Liu , S. Liu , K. Deng , E. Kan , J. Phys. Chem. C 2015, 119, 6923.

[69] H. Liu , A. T. Neal , Z. Zhu , D. Tomanek , P. D. Ye , 2014, arXiv:1401.4133.

[70] J.‐W. Jiang , Nanotechnology 2015, 26, 055701. 25571869 10.1088/0957-4484/26/5/055701

[71] K. F. Mak , C. Lee , J. Hone , J. Shan , T. F. Heinz , Phys. Rev. Lett. 2010, 105, 136805. 21230799 10.1103/PhysRevLett.105.136805

[72] Q. Wei , X. Peng , Appl. Phys. Lett. 2014, 104, 251915.

[73] L. Li , G. J. Ye , V. Tran , R. Fei , G. Chen , H. Wang , J. Wang , K. Watanabe , T. Taniguchi , L. Yang , Nat. Nanotechnol. 2015, 10, 608. 25984835 10.1038/nnano.2015.91

[74] E. Flores , J. R. Ares , A. Castellanos‐Gomez , M. Barawi , I. J. Ferrer , C. Sánchez , Appl. Phys. Lett. 2015, 106, 022102.

[75] L. Seixas , A. Rodin , A. Carvalho , A. C. Neto , Phys. Rev. B 2015, 91, 115437.

[76] A. Rodin , A. Carvalho , A. C. Neto , Phys. Rev. Lett. 2014, 112, 176801. 24836264 10.1103/PhysRevLett.112.176801

[77] D. W. Drumm , M. C. Per , A. Budi , L. C. Hollenberg , S. P. Russo , Nanoscale Res. Lett. 2014, 9, 1. 25246862 10.1186/1556-276X-9-443 PMC4158386

[78] J. Dai , X. C. Zeng , J. Phys. Chem. Lett. 2014, 5, 1289. 26274486 10.1021/jz500409m

[79] E. S. Reich , Nature 2014, 506, 19. 24499900 10.1038/506019a

[80] Q. Tang , Z. Zhou , Prog. Mater. Sci. 2013, 58, 1244.

[81] Y. Du , H. Liu , Y. Deng , P. D. Ye , ACS Nano 2014, 8, 10035. 25314022 10.1021/nn502553m

[82] S. Das , M. Demarteau , A. Roelofs , ACS Nano 2014, 8, 11730. 25329532 10.1021/nn505868h

[83] J.‐S. Kim , Y. Liu , W. Zhu , S. Kim , D. Wu , L. Tao , A. Dodabalapur , K. Lai , D. Akinwande , Sci. Rep. 2015, 5, 8989. 25758437 10.1038/srep08989 PMC4355728

[84] H. Yuan , X. Liu , F. Afshinmanesh , W. Li , G. Xu , J. Sun , B. Lian , G. Ye , Y. Hikita , Z. Shen , 2014, arXiv:1409.4729.

[85] L. Kong , Z. Qin , G. Xie , Z. Guo , H. Zhang , P. Yuan , L. Qian , 2015, arXiv:1508.04510.≈

[86] M. Buscema , D. J. Groenendijk , S. I. Blanter , G. A. Steele , H. S. van der Zant , A. Castellanos‐Gomez , Nano Lett. 2014, 14, 3347. 24821381 10.1021/nl5008085

[87] S. Zhao , W. Kang , J. Xue , J. Mater. Chem. A 2014, 2, 19046.

[88] Z. T. Wang , Y. Xu , Z. Guo , C. Zhao , H. Zhang , Conference on Lasers and Electro‐Optics: OSA Technical Digest, Optical Society of America: p STu1R‐2 2016.

[89] Z. Wang , Y. Xu , S. C. Dhanabalan , J. S. Ponraj , C. Zhao , C. Xu , Y. Xiang , J. Li , H. Zhang , IEEE Photonics J. 2016, 99, 1.

[90] Y. Wang , G. Huang , H. Mu , S. Lin , J. Chen , S. Xiao , Q. Bao , J. He , Appl. Phys. Lett. 2015, 107, 091905.

[91] Z.‐C. Luo , M. Liu , Z.‐N. Guo , X.‐F. Jiang , A.‐P. Luo , C.‐J. Zhao , X.‐F. Yu , W.‐C. Xu , H. Zhang , Opt. Express 2015, 23, 20030. 26367661 10.1364/OE.23.020030

[92] J. Li , H. Luo , B. Zhai , R. Lu , Z. Guo , H. Zhang , Y. Liu , Sci. Rep. 2016, 6, 30361. 27457338 10.1038/srep30361 PMC4960592

[93] H. Mu , S. Lin , Z. Wang , S. Xiao , P. Li , Y. Chen , H. Zhang , H. Bao , S. P. Lau , C. Pan , Adv. Opt. Mater. 2015, 3, 1447.

[94] Z. Luo , D. Wu , B. Xu , H. Xu , Z. Cai , J. Peng , J. Weng , S. Xu , C. Zhu , F. Wang , Nanoscale 2016, 8, 1066. 26658877 10.1039/c5nr06981e

[95] J. Ma , S. Lu , Z. Guo , X. Xu , H. Zhang , D. Tang , D. Fan , Opt. Express 2015, 23, 22643. 26368232 10.1364/OE.23.022643

[96] L. Kou , T. Frauenheim , C. Chen , J. Phys. Chem. Lett. 2014, 5, 2675. 26277962 10.1021/jz501188k

[97] D. Li , H. Jussila , L. Karvonen , G. Ye , H. Lipsanen , X. Chen , Z. Sun , 2015, arXiv:1505.00480. 10.1038/srep15899 PMC4626849 26514090

[98] J. Sotor , G. Sobon , W. Macherzynski , P. Paletko , K. Grodecki , K. M. Abramski , Opt. Mater. Express 2014, 4, 1.

[99] Q. Bao , H. Zhang , Y. Wang , Z. Ni , Y. Yan , Z. X. Shen , K. P. Loh , D. Y. Tang , Adv. Funct. Mater. 2009, 19, 3077.

[100] Z. Sun , T. Hasan , F. Torrisi , D. Popa , G. Privitera , F. Wang , F. Bonaccorso , D. M. Basko , A. C. Ferrari , ACS Nano 2010, 4, 803. 20099874 10.1021/nn901703e

[101] U. Keller , K. J. Weingarten , F. X. Kartner , D. Kopf , B. Braun , I. D. Jung , R. Fluck , C. Honninger , N. Matuschek , J. A. Der Au , IEEE J. Sel. Top. Quantum Electron. 1996, 2, 435.

[102] F. Wang , A. Rozhin , V. Scardaci , Z. Sun , F. Hennrich , I. White , W. I. Milne , A. C. Ferrari , Nat. Nanotechnol. 2008, 3, 738. 19057594 10.1038/nnano.2008.312

[103] P. Bridgman , J. Am. Chem. Soc. 1914, 36, 1344.

[104] P. W. Bridgman , Phys. Rev. 1914, 3, 153.

[105] Z. Wang , H. Jia , X. Zheng , R. Yang , Z. Wang , G. Ye , X. Chen , J. Shan , P. X.‐L. Feng , Nanoscale 2015, 7, 877. 25385657 10.1039/c4nr04829f

[106] S. Endo , Y. Akahama , S.‐i. Terada , S.‐i. Narita , Jpn. J. Appl. Phys. 1982, 21, L482.

[107] L.‐Q. Sun , M.‐J. Li , K. Sun , S.‐H. Yu , R.‐S. Wang , H.‐M. Xie , J. Phys. Chem. C 2012, 116, 14772.

[108] D. Li , H. Jussila , L. Karvonen , G. Ye , H. Lipsanen , X. Chen , Z. Sun , Sci. Rep. 2015, 5, 15899. 26514090 10.1038/srep15899 PMC4626849

[109] M. Baba , F. Izumida , Y. Takeda , A. Morita , Jpn. J. Appl. Phys. 1989, 28, 1019.

[110] Z. Sofer , D. Bouša , J. Luxa , V. Mazanek , M. Pumera , Chem. Commun. 2016, 52, 1563. 10.1039/c5cc09150k 26691583

[111] L. Wang , Z. Sofer , M. Pumera , ChemElectroChem 2015, 2, 324.

[112] T. Nilges , M. Kersting , T. Pfeifer , J. Solid State Chem. 2008, 181, 1707.

[113] S. Lange , P. Schmidt , T. Nilges , Inorg. Chem. 2007, 46, 4028. 17439206 10.1021/ic062192q

[114] H. Wang , X. Yang , W. Shao , S. Chen , J. Xie , X. Zhang , J. Wang , Y. Xie , J. Am. Chem. Soc. 2015, 137, 11376. 26284535 10.1021/jacs.5b06025

[115] M. Nagao , A. Hayashi , M. Tatsumisago , J. Power Sources 2011, 196, 6902.

[116] J. Sun , G. Zheng , H.‐W. Lee , N. Liu , H. Wang , H. Yao , W. Yang , Y. Cui , Nano Lett. 2014, 14, 4573. 25019417 10.1021/nl501617j

[117] I. Shirotani , Mol. Cryst. Liq. Cryst. 1982, 86, 203.

[118] X. Zhang , H. Xie , Z. Liu , C. Tan , Z. Luo , H. Li , J. Lin , L. Sun , W. Chen , Z. Xu , Angew. Chem. Int. Ed. 2015, 54, 3653. 10.1002/anie.201409400 25649505

[119] Y. Maruyama , S. Suzuki , T. Osaki , H. Yamaguchi , S. Sakai , K. Nagasato , I. Shirotani , Bull. Chem. Soc. Jpn 1986, 59, 1067.

[120] N. Iwasaki , Y. Maruyama , S. Kurihara , I. Shirotani , M. Kinoshita , Chem. Lett. 1985, 1, 119.

[121] S. Ge , C. Li , Z. Zhang , C. Zhang , Y. Zhang , J. Qiu , Q. Wang , J. Liu , S. Jia , J. Feng , Nano Lett. 2015, 15, 4650. 26039361 10.1021/acs.nanolett.5b01409

[122] M. Köpf , N. Eckstein , D. Pfister , C. Grotz , I. Krüger , M. Greiwe , T. Hansen , H. Kohlmann , T. Nilges , J. Cryst. Growth 2014, 405, 6.

[123] S. Lee , F. Yang , J. Suh , S. Yang , Y. Lee , G. Li , H. S. Choe , A. Suslu , Y. Chen , C. Ko , Nat. Commun. 2015, 6, 8573. 26472285 10.1038/ncomms9573 PMC4634207

[124] S. Lange , T. Nilges , Z. Naturforsch. B 2006, 61, 871.

[125] Y. Oumellal , A. Rougier , J.‐M. Tarascon , L. Aymard , J. Power Sources 2009, 192, 698.

[126] V. Sresht , A. A. Pádua , D. Blankschtein , ACS Nano 2015, 9, 8255. 26192620 10.1021/acsnano.5b02683

[127] Y. Saito , Y. Iwasa , ACS Nano 2015, 9, 3192. 25712777 10.1021/acsnano.5b00497

[128] Y. Anugrah , M. C. Robbins , P. A. Crowell , S. J. Koester , Appl. Phys. Lett. 2015, 106, 103108.

[129] J. Yang , R. Xu , J. Pei , Y. W. Myint , F. Wang , Z. Wang , S. Zhang , Z. Yu , Y. Lu , 2014, arXiv:1412.6701.

[130] K. S. Novoselov , A. K. Geim , S. Morozov , D. Jiang , Y. Zhang , S. a. Dubonos , I. Grigorieva , A. Firsov , Science 2004, 306, 666. 15499015 10.1126/science.1102896

[131] R. Frindt , J. Appl. Phys. 1966, 37, 1928.

[132] K. S. Novoselov , V. Fal , L. Colombo , P. Gellert , M. Schwab , K. Kim , Nature 2012, 490, 192. 23060189 10.1038/nature11458

[133] A. K. Geim , Science 2009, 324, 1530. 19541989 10.1126/science.1158877

[134] K. Novoselov , D. Jiang , F. Schedin , T. Booth , V. Khotkevich , S. Morozov , A. Geim , Proc. Natl. Acad. Sci. U. S. A. 2005, 102, 10451. 16027370 10.1073/pnas.0502848102 PMC1180777

[135] V. Nicolosi , M. Chhowalla , M. G. Kanatzidis , M. S. Strano , J. N. Coleman , Science 2013, 340, 1226419.

[136] A. K. Geim , K. S. Novoselov , Nat. Mater. 2007, 6, 183. 17330084 10.1038/nmat1849

[137] J. C. Meyer , A. K. Geim , M. Katsnelson , K. Novoselov , T. Booth , S. Roth , Nature 2007, 446, 60. 17330039 10.1038/nature05545

[138] C. Huo , Z. Yan , X. Song , H. Zeng , Sci. Bull. 2015, 60, 1994.

[139] M. J. Allen , V. C. Tung , R. B. Kaner , Chem. Rev. 2009, 110, 132. 10.1021/cr900070d 19610631

[140] X. Ling , H. Wang , S. Huang , F. Xia , M. S. Dresselhaus , Proc. Natl. Acad. Sci. 2015, 112, 4523. 25820173 10.1073/pnas.1416581112 PMC4403146

[141] F. Bachhuber , J. von Appen , R. Dronskowski , P. Schmidt , T. Nilges , A. Pfitzner , R. Weihrich , Angew. Chem. Int. Ed. 2014, 53, 11629. 10.1002/anie.201404147 25196550

[142] W. Gao , A. Tkatchenko , Phys. Rev. Lett. 2015, 114, 096101. 25793829 10.1103/PhysRevLett.114.096101

[143] G. Wang , D. Yang , Z. Zhang , M. Si , D. Xue , H. He , R. Pandey , Appl. Phys. Lett. 2014, 105, 121605.

[144] N. Marom , J. Bernstein , J. Garel , A. Tkatchenko , E. Joselevich , L. Kronik , O. Hod , Phys. Rev. Lett. 2010, 105, 046801. 20867872 10.1103/PhysRevLett.105.046801

[145] Z. Zhu , D. Tománek , Phys. Rev. Lett. 2014, 112, 176802. 24836265 10.1103/PhysRevLett.112.176802

[146] J. Xie , M. Si , D. Yang , Z. Zhang , D. Xue , J. Appl. Phys. 2014, 116, 073704.

[147] J. P. Oviedo , S. KC , N. Lu , J. Wang , K. Cho , R. M. Wallace , M. J. Kim , ACS Nano 2014, 9, 1543. 25494557 10.1021/nn506052d

[148] Z. Luo , J. Maassen , Y. Deng , Y. Du , R. P. Garrelts , M. S. Lundstrom , D. Y. Peide , X. Xu , Nat. Commun. 2015, 6, 8572. 26472191 10.1038/ncomms9572 PMC4634212

[149] S. Sabri , P. Levesque , C. Aguirre , J. Guillemette , R. Martel , T. Szkopek , Appl. Phys. Lett. 2009, 95, 242104.

[150] A. Favron , E. Gaufrès , F. Fossard , A.‐L. Phaneuf‐L'Heureux , N. Y. Tang , P. L. Lévesque , A. Loiseau , R. Leonelli , S. Francoeur , R. Martel , Nat. Mater. 2015, 14, 826. 26006004 10.1038/nmat4299

[151] X. Liu , J. D. Wood , K.‐S. Chen , E. Cho , M. C. Hersam , J Phys. Chem. Lett. 2015, 6, 773. 26262651 10.1021/acs.jpclett.5b00043

[152] R. Xu , S. Zhang , F. Wang , J. Yang , Z. Wang , J. Pei , Y. W. Myint , B. Xing , Z. Yu , L. Fu , ACS Nano 2015. 10.1021/acsnano.5b06193 26713882

[153] H. Li , Q. Zhang , C. C. R. Yap , B. K. Tay , T. H. T. Edwin , A. Olivier , D. Baillargeat , Adv. Funct. Mater. 2012, 22, 1385.

[154] J. Yang , R. Xu , J. Pei , Y. W. Myint , F. Wang , Z. Wang , S. Zhang , Z. Yu , Y. Lu , Light: Sci. Appl. 2015, 4, e312.

[155] J. O. Island , G. A. Steele , H. S. van der Zant , A. Castellanos‐Gomez , 2D Mater. 2015, 2, 011002.

[156] A. Castellanos‐Gomez , L. Vicarelli , E. Prada , J. O. Island , K. Narasimha‐Acharya , S. I. Blanter , D. J. Groenendijk , M. Buscema , G. A. Steele , J. Alvarez , 2D Mater. 2014, 1, 025001.

[157] A. Castellanos‐Gomez , M. Buscema , R. Molenaar , V. Singh , L. Janssen , H. S. van der Zant , G. A. Steele , 2D Mater. 2014, 1, 011002.

[158] M. A. Meitl , Z.‐T.‐ Zhu , V. Kumar , K. J. Lee , X. Feng , Y. Y. Huang , I. Adesida , R. G. Nuzzo , J. A. Rogers , Nat. Mater. 2006, 5, 33.

[159] D. R. Dreyer , S. Park , C. W. Bielawski , R. S. Ruoff , Chem. Soc. Rev. 2010, 39, 228. 20023850 10.1039/b917103g

[160] J. N. Coleman , Acc. Chem. Res. 2012, 46, 14. 22433117 10.1021/ar300009f

[161] G. Walker , W. Garrett , Science 1967, 156, 385. 17812385 10.1126/science.156.3773.385

[162] P. Joensen , R. Frindt , S. R. Morrison , Mater. Res. Bull. 1986, 21, 457.

[163] D. Murphy , G. Hull Jr ., J. Chem. Phys. 1975, 62, 973.

[164] C. Liu , O. Singh , P. Joensen , A. Curzon , R. Frindt , Thin Solid Films 1984, 113, 165.

[165] M. M. J. Treacy , S. Rice , A. J. Jacobson , J. Lewandowski , Chem. Mater. 1990, 2, 279.

[166] J. N. Coleman , M. Lotya , A. O'Neill , S. D. Bergin , P. J. King , U. Khan , K. Young , A. Gaucher , S. De , R. J. Smith , Science 2011, 331, 568. 21292974 10.1126/science.1194975

[167] G. Eda , M. Chhowalla , Adv. Mater. 2010, 22, 2392. 20432408 10.1002/adma.200903689

[168] C.‐J. Shih , A. Vijayaraghavan , R. Krishnan , R. Sharma , J.‐H. Han , M.‐H. Ham , Z. Jin , S. Lin , G. L. Paulus , N. F. Reuel , Nat. Nanotechnol. 2011, 6, 439. 21706026 10.1038/nnano.2011.94

[169] G. Eda , H. Yamaguchi , D. Voiry , T. Fujita , M. Chen , M. Chhowalla , Nano Lett. 2011, 11, 5111. 22035145 10.1021/nl201874w

[170] G. Eda , T. Fujita , H. Yamaguchi , D. Voiry , M. Chen , M. Chhowalla , Acs Nano 2012, 6, 7311. 22799455 10.1021/nn302422x

[171] R. Ma , T. Sasaki , Adv. Mater. 2010, 22, 5082. 20925100 10.1002/adma.201001722

[172] T. Tanaka , Y. Ebina , K. Takada , K. Kurashima , T. Sasaki , Chem. Mater. 2003, 15, 3564.

[173] L. Chen , G. Zhou , Z. Liu , X. Ma , J. Chen , Z. Zhang , X. Ma , F. Li , H. M. Cheng , W. Ren , Adv. Mater. 2016, 28, 510. 26584241 10.1002/adma.201503678

[174] Y. Hernandez , M. Lotya , D. Rickard , S. D. Bergin , J. N. Coleman , Langmuir 2009, 26, 3208. 10.1021/la903188a 19883090

[175] A. F. Barton , CRC handbook of solubility parameters and other cohesion parameters, CRC Press, Boca Rotan, USA 1991.

[176] C. M. Hansen , Hansen solubility parameters: a user's handbook, CRC Press, Boca Rotan, USA, 2007.

[177] X. Zhang , X. Xie , H. Wang , J. Zhang , B. Pan , Y. Xie , J. Am. Chem. Soc. 2012, 135, 18. 23244197 10.1021/ja308249k

[178] C.‐J. Shih , S. Lin , M. S. Strano , D. Blankschtein , J. Am. Chem. Soc. 2010, 132, 14638. 20879739 10.1021/ja1064284

[179] G. Kamath , G. A. Baker , RSC Adv. 2013, 3, 8197.

[180] G. Kamath , G. A. Baker , Phys. Chem. Chem. Phys. 2012, 14, 7929. 22552225 10.1039/c2cp40824d

[181] T. Ludwig , L. Guo , P. McCrary , Z. Zhang , H. Gordon , H. Quan , M. Stanton , R. M. Frazier , R. D. Rogers , H.‐T. Wang , Langmuir 2015, 31, 3644. 25760309 10.1021/acs.langmuir.5b00239

[182] P. Yasaei , B. Kumar , T. Foroozan , C. Wang , M. Asadi , D. Tuschel , J. E. Indacochea , R. F. Klie , A. Salehi‐Khojin , Adv. Mater. 2015, 27, 1887. 25645510 10.1002/adma.201405150

[183] J. Kang , J. D. Wood , S. A. Wells , J.‐H. Lee , X. Liu , K.‐S. Chen , M. C. Hersam , ACS Nano 2015, 9, 3596. 25785299 10.1021/acsnano.5b01143

[184] L. Niu , J. N. Coleman , H. Zhang , H. Shin , M. Chhowalla , Z. Zheng , Small 2016, 12, 272. 26663877 10.1002/smll.201502207

[185] X. Zheng , R. Chen , G. Shi , J. Zhang , Z. Xu , T. Jiang , Opt. Lett. 2015, 40, 3480. 26258337 10.1364/OL.40.003480

[186] J. R. Brent , N. Savjani , E. A. Lewis , S. J. Haigh , D. J. Lewis , P. O'Brien , Chem. Commun. 2014, 50, 13338. 10.1039/c4cc05752j 25231502

[187] J. Sun , H.‐W. Lee , M. Pasta , H. Yuan , G. Zheng , Y. Sun , Y. Li , Y. Cui , Nat. Nanotechnol 2015, 10, 980. 26344183 10.1038/nnano.2015.194

[188] A. O'Neill , U. Khan , P. N. Nirmalraj , J. Boland , J. N. Coleman , J. Phys. Chem. C 2011, 115, 5422.

[189] M. Lotya , Y. Hernandez , P. J. King , R. J. Smith , V. Nicolosi , L. S. Karlsson , F. M. Blighe , S. De , Z. Wang , I. McGovern , J. Am. Chem. Soc. 2009, 131, 3611. 19227978 10.1021/ja807449u

[190] P. May , U. Khan , A. O'Neill , J. N. Coleman , J. Mater. Chem. 2012, 22, 1278.

[191] D. Hanlon , C. Backes , E. Doherty , C. S. Cucinotta , N. C. Berner , C. Boland , K. Lee , A. Harvey , P. Lynch , Z. Gholamvand , S. Zhang , K. Wang , G. Moynihan , A. Pokle , Q. M. Ramasse , N. McEvoy , W. J. Blau , J. Wang , G. Abellan , F. Hauke , A. Hirsch , S. Sanvito , D. D. O'Regan , G. S. Duesberg , V. Nicolosi , J. N. Coleman , Nat. Commun. 2015, 6, 8563. 26469634 10.1038/ncomms9563 PMC4634220

[192] K. R. Paton , E. Varrla , C. Backes , R. J. Smith , U. Khan , A. O'Neill , C. Boland , M. Lotya , O. M. Istrate , P. King , Nat. Mater. 2014, 13, 624. 24747780 10.1038/nmat3944

[193] C. Backes , R. J. Smith , N. McEvoy , N. C. Berner , D. McCloskey , H. C. Nerl , A. O'Neill , P. J. King , T. Higgins , D. Hanlon , Nat. Commun. 2014, 5, 4576. 25099520 10.1038/ncomms5576

[194] U. Khan , A. O'Neill , H. Porwal , P. May , K. Nawaz , J. N. Coleman , Carbon 2012, 50, 470.

[195] P. Yasaei , A. Behranginia , T. Foroozan , M. Asadi , K. Kim , F. Khalili‐Araghi , A. Salehi‐Khojin , ACS Nano 2015, 9, 9898. 26401950 10.1021/acsnano.5b03325

[196] A. H. Woomer , T. W. Farnsworth , J. Hu , R. A. Wells , C. L. Donley , S. C. Warren , ACS Nano 2015, 9, 8869. 26256770 10.1021/acsnano.5b02599

[197] H. U. Lee , S. Y. Park , S. C. Lee , S. Choi , S. Seo , H. Kim , J. Won , K. Choi , K. S. Kang , H. G. Park , Small 2016, 12, 214. 26584654 10.1002/smll.201502756

[198] P. Hapiot , C. Lagrost , Chem. Rev. 2008, 108, 2238. 18564878 10.1021/cr0680686

[199] Y. Cao , T. Mu , Ind. Eng. Chem. Res. 2014, 53, 8651.

[200] W. Zhao , Z. Xue , J. Wang , J. Jiang , X. Zhao , T. Mu , ACS Appl. Mater. Interfaces 2015, 7, 27608. 26642883 10.1021/acsami.5b10734

[201] X. Wang , P. F. Fulvio , G. A. Baker , G. M. Veith , R. R. Unocic , S. M. Mahurin , M. Chi , S. Dai , Chem. Commun. 2010, 46, 4487. 10.1039/c0cc00799d 20485780

[202] T. Morishita , H. Okamoto , Y. Katagiri , M. Matsushita , K. Fukumori , Chem. Commun. 2015, 51, 12068. 10.1039/c5cc04077a 26121635

[203] W. Zhang , Y. Wang , D. Zhang , S. Yu , W. Zhu , J. Wang , F. Zheng , S. Wang , J. Wang , Nanoscale 2015, 7, 10210. 25990823 10.1039/c5nr02253c

[204] M. Tariq , M. G. Freire , B. Saramago , J. A. Coutinho , J. N. C. Lopes , L. P. N. Rebelo , Chem. Soc. Rev. 2012, 41, 829. 21811714 10.1039/c1cs15146k

[205] Z. Sun , H. Xie , S. Tang , X. F. Yu , Z. Guo , J. Shao , H. Zhang , H. Huang , H. Wang , P. K. Chu , Angew. Chem. 2015, 127, 11688. 10.1002/anie.201506154 26296530

[206] E. Varrla , K. R. Paton , C. Backes , A. Harvey , R. J. Smith , J. McCauley , J. N. Coleman , Nanoscale 2014, 6, 11810. 25164103 10.1039/c4nr03560g

[207] F. Xu , B. Ge , J. Chen , A. Nathan , L. L Xin , H. Ma , H. Min, C. Zhu , W. Xia , Z. Li , S. Li , K. Yu , L. Wu , Y. Cui , L. Sun , Y. Zhu , 2D Mater. 2016, 3, 025005.

[208] J. M. Tour , Nat. Mater. 2014, 13, 545. 24747779 10.1038/nmat3961

[209] Y. Liu , H. Nan , X. Wu , W. Pan , W. Wang , J. Bai , W. Zhao , L. Sun , X. Wang , X. Ni , Z. , ACS Nano 2013, 7, 4202. 23548109 10.1021/nn400644t

[210] A. Castellanos‐Gomez , M. Barkelid , A. Goossens , V. E. Calado , H. S. van der Zant , G. A. Steele , Nano Lett. 2012, 12, 3187. 22642212 10.1021/nl301164v

[211] W. Lu , H. Nan , J. Hong , Y. Chen , C. Zhu , Z. Liang , X. Ma , Z. Ni , C. Jin , Z. Zhang , Nano Res. 2014, 7, 853.

[212] J. Jia , S. K. Jang , S. Lai , J. Xu , Y. J. Choi , J.‐H. Park , S. Lee , ACS Nano 2015, 9, 8729. 26301840 10.1021/acsnano.5b04265

[213] Z. Yang , J. Hao , S. Yuan , S. Lin , H. M. Yau , J. Dai , S. P. Lau , Adv. Mater. 2015, 27, 3748. 25973767 10.1002/adma.201500990

[214] A. Molle , C. Grazianetti , D. Chiappe , E. Cinquanta , E. Cianci , G. Tallarida , M. Fanciulli , Adv. Funct. Mater. 2013, 23, 4340.

[215] R. Lieth , Preparation and crystal growth of materials with layered structures, Volume 1, D. Riedel Pblishing, Dordrecht, The Netherlands, 1977.

[216] S.‐L. Yau , T. P. Moffat , A. J. Bard , Z. Zhang , M. M. Lerner , Chem. Phys. Lett. 1992, 198, 383.

[217] A. Javey , H. Kim , M. Brink , Q. Wang , A. Ural , J. Guo , P. McIntyre , P. McEuen , M. Lundstrom , H. Dai , Nat. Mater. 2002, 1, 241. 12618786 10.1038/nmat769

[218] V. K. Sangwan , D. Jariwala , K. Everaerts , J. J. McMorrow , J. He , M. Grayson , L. J. Lauhon , T. J. Marks , M. C. Hersam , Appl. Phys. Lett. 2014, 104, 083503.

[219] S. Kim , J. Nah , I. Jo , D. Shahrjerdi , L. Colombo , Z. Yao , E. Tutuc , S. K. Banerjee , 2009, arXiv:0901.2901.

[220] H. Liu , A. T. Neal , M. Si , Y. Du , P. D. Ye , IEEE Electron Device Lett. 2014, 35, 795.

[221] K. L. Utt , P. Rivero , M. Mehboudi , E. O. Harriss , M. F. Borunda , A. Pacheco SanJuan , S. Barraza‐Lopez , ACS Cent. Sci. 2015, 1, 320. 27162987 10.1021/acscentsci.5b00244 PMC4827457

[222] G. Wang , W. J. Slough , R. Pandey , S. P. Karna , 2015, arXiv:1508.07461.

[223] A. Ziletti , A. Carvalho , D. K. Campbell , D. F. Coker , A. C. Neto , Phys. Rev. Lett. 2015, 114, 046801. 25679901 10.1103/PhysRevLett.114.046801

[224] G. Wang , R. Pandey , S. P. Karna , Nanoscale 2015, 7, 524. 25412501 10.1039/c4nr05384b

[225] B. Sa , Y.‐L. Li , J. Qi , R. Ahuja , Z. Sun , J. Phys. Chem. C 2014, 118, 26560.

[226] Y. Cai , Q. Ke , G. Zhang , Y.‐W. Zhang , J. Phys. Chem. C 2015, 119, 3102.

[227] J. H. Han , S. Lee , D. Yoo , J.‐H. Lee , S. Jeong , J.‐G. Kim , J. Cheon , J. Am. Chem. Soc. 2013, 135, 3736. 23458758 10.1021/ja309744c

[228] J. Brunner , M. Thüler , S. Veprek , R. Wild , J. Phys. Chem. Solids 1979, 40, 967.

[229] C. Sendner , D. Horinek , L. Bocquet , R. R. Netz , Langmuir 2009, 25, 10768. 19591481 10.1021/la901314b

[230] R. A. Doganov , E. C. O'Farrell , S. P. Koenig , Y. Yeo , A. Ziletti , A. Carvalho , D. K. Campbell , D. F. Coker , K. Watanabe , T. Taniguchi , Nat. Commun. 2015, 6, 6647. 25858614 10.1038/ncomms7647

[231] L. Britnell , R. V. Gorbachev , R. Jalil , B. D. Belle , F. Schedin , M. I. Katsnelson , L. Eaves , S. V. Morozov , A. S. Mayorov , N. M. Peres , Nano Lett. 2012, 12, 1707. 22380756 10.1021/nl3002205

[232] J. S. Bunch , S. S. Verbridge , J. S. Alden , A. M. Van Der Zande , J. M. Parpia , H. G. Craighead , P. L. McEuen , Nano Lett. 2008, 8, 2458. 18630972 10.1021/nl801457b

[233] S. P. Koenig , L. Wang , J. Pellegrino , J. S. Bunch , Nat. Nanotechnol. 2012, 7, 728. 23042491 10.1038/nnano.2012.162

[234] J. Kim , S. K. Baek , K. S. Kim , Y. J. Chang , E. Choi , Curr. Appl. Phys. 2016, 16, 165.

[235] H. U. Lee , S. C. Lee , J. Won , B.‐C. Son , S. Choi , Y. Kim , S. Y. Park , H.‐S. Kim , Y.‐C‐ Lee , J. Lee , Sci. Rep. 2015, 5, 8691. 25732720 10.1038/srep08691 PMC4346807

[236] B. Wan , B. Yang , Y. Wang , J. Zhang , Z. Zeng , Z. Liu , W. Wang , Nanotechnology 2015, 26, 435702. 26436439 10.1088/0957-4484/26/43/435702

[237] Y. Du , C. Ouyang , S. Shi , M. Lei , J. Appl. Phys. 2010, 107, 093718.

[238] Y. Huang , J. Qiao, K. He , S. Bliznakov , E. Sutter , X. Chen , D. Luo , F. Meng , D. Su , J. Decker , W. Ji , R. S. Ruoff , P. Sutter , Chem. Mater., 2016, 28, 8330.

[239] B. Zhang , F. Lou , R. Zhao , J. He , J. Li , X. Su , J. Ning , K. Yang , Opt. Lett. 2015, 40, 3691. 26274636 10.1364/OL.40.003691

[240] X. Wang , A. M. Jones , K. L. Seyler , V. Tran , Y. Jia , H. Zhao , H. Wang , L. Yang , X. Xu , F. Xia , Nat. Nanotechnol. 2015, 10, 517. 25915195 10.1038/nnano.2015.71

[241] D. Hanlon , C. Backes , E. Doherty , C. S. Cucinotta , N. C. Berner , C. Boland , K. Lee , A. Harvey , P. Lynch , Z. Gholamvand , Nat. Commun. 2015, 6, 8563. 26469634 10.1038/ncomms9563 PMC4634220

